# Valorization of *Amphidinium carterae* for Integrated Preparation of Peridinin and Diadinoxanthin Cycle Carotenoids

**DOI:** 10.3390/md23100405

**Published:** 2025-10-17

**Authors:** Yi Li, Gengjie Zhuang, Xuan Zhang, Wei Cui, Zhiwei Hong, Jianhua Fan, Jinrong Zhang, Xiaojun Yan

**Affiliations:** 1 College of Food Sciences and Engineering, Ningbo University, Ningbo 315211, China; liyi20241205@163.com (Y.L.); 19857990154@163.com (X.Z.); 2 School of Marine Sciences, Ningbo University, Ningbo 315211, China; zhuanggengjie123@163.com; 3 Health Science Center, Ningbo University, Ningbo 315211, China; cuiwei@nbu.edu.cn; 4 State Key Laboratory of Bioreactor Engineering, East China University of Science and Technology, Shanghai 200237, China; y30230492@mail.ecust.edu.cn (Z.H.); jhfan@ecust.edu.cn (J.F.); 5 Department of Applied Biology, East China University of Science and Technology, Shanghai 200237, China; 6 Key Laboratory of Applied Marine Biotechnology of Ministry of Education, Ningbo University, Ningbo 315211, China

**Keywords:** integrated biorefinery, microalgae, extraction, purification, diatoxanthin

## Abstract

An integrated microalgae biorefinery producing high-purity xanthophylls using a sustainable and efficient strategy still faces critical challenges. In this study, the microalga *Amphidinium carterae* can accumulate peridinin and diadinoxanthin cycle carotenoids. Notably, valorization of wet *A. carterae* using integrated preparation of peridinin and diadinoxanthin cycle carotenoids was developed, containing four main steps including microalgae cultivation, solvent extraction, octadecylsilyl open-column chromatography, and ethanol precipitation for the first time. Under the optimum integrated preparation conditions, the purities of obtained peridinin, diadinoxanthin, and diatoxanthin were all more than 95%, with total recovery rates of approximately 70%, 51%, and 74%, respectively. Based on nuclear magnetic resonance techniques, the purified peridinin, diadinoxanthin, and diatoxanthin were identified as all-*trans*-peridinin, all-*trans*-diadinoxanthin, and all-*trans*-diatoxanthin, respectively. In all, the developed method may hold significant implications for future purification of peridinin and diadinoxanthin cycle carotenoids, as well as for the integrated biorefinery of wet *A. carterae*.

## 1. Introduction

Microalgae have been regarded as the most promising and sustainable feedstock for diverse high-value products [1]. Multiproduct biorefinery is supposed to be an economically feasible strategy for developing the microalgae industry [2].

Peridinin (PER), a characteristic carotenoid of dinoflagellates, possesses various beneficial biological effects, including antilipoperoxidant, anti-inflammatory, and anti-cancer [3,4,5]. Currently, PER synthesis methods have been reported [6]. The diadinoxanthin cycle (DD-cycle), involving two xanthophyll components, including diadinoxanthin (DDX) and diatoxanthin (DTX), represents a vital photoprotective mechanism for some microalgae [7]. DDX possesses antioxidant and antibacterial activities [8]. DTX has diverse beneficial biological effects, including antioxidant and anti-inflammatory [9,10].

Due to the promising applications of PER and DD-cycle carotenoids, various preparation methods have been developed. However, the industrial preparation of PER, DDX, and DTX still faces challenges due to some unignorable factors, including the chemically unstable nature of the three carotenoid pigments, the lack of high-quality raw materials, and the lack of environmentally friendly preparation methods. Several methods have been established for the isolation of PER from dinoflagellates, including high-performance liquid chromatography (HPLC) and preparative circular chromatography (Chromatotron) [11,12]. Some methods have been investigated for the integrated purification of DDX and DTX, including silica gel column chromatography (SGCC) and HPLC [13]. However, the above preparation methods are limited due to the use of toxic reagents and high production costs. Notably, the simultaneous preparation of PER and DD-cycle carotenoids from microalgae feedstocks has not yet been reported.

The discovery of high-quality microalgae feedstocks is crucial for the integrated preparation of the three carotenoids (PER and DD-cycle carotenoids). *Amphidinium carterae*, a worldwide distributed dinoflagellate, can cause the formation of harmful algal blooms when it coexists with other microalgae [14,15]. Notably, *A. carterae* is also renowned because it is a high-biomass producer, as well as a producer of diverse high-value products, such as carotenoids and fatty acids [16]. *A. carterae* can be cultured successfully on a large scale in diverse modes, including closed systems and semi-continuous modes in indoor and outdoor environments. Some progress has been made in the outdoor cultivation of *A. carterae* on a large scale for the co-production of unsaturated fatty acids and carotenoids [17]. Additionally, the co-extraction of three types of biocompounds (fatty acids, amphidinols, and carotenoids) from lyophilized *A. carterae* was explored using solvent extraction [16,18]. *A. carterae* was potentially a microalgal feedstock for the co-production of PER and DD-cycle carotenoids [19].

An effective method for the simultaneous preparation of the three carotenoids should be species-specific, which is established based on the particular microalgal feedstock and the physicochemical properties of the target carotenoids [20].The simultaneous preparation of PER and DD-cycle carotenoids from the biomass of *A. carterae* still faces great challenges due to some reasons, including the chemical composition profile of *A. carterae,* the structural components of the microalgal cell wall, the chemically unstable nature of carotenoids, and the differences in extraction conditions [21]. In terms of economic feasibility, the integrated preparation of the three carotenoids could be more profitable for the valorization of microalgal biomass [22]. Therefore, developing an effective and green protocol for the simultaneous preparation of PER and DD-cycle carotenoids from *A. carterae* is vital for further valorization of the microalgal biomass.

Ethanol extraction is commonly used to extract a variety of high-value products in the food and pharmaceutical industries [23]. Octadecylsilyl (ODS) column chromatography is generally applied to purify multiple active compounds [24]. Ethanol precipitation is also widely used to refine various compounds in the food, cosmetics, and pharmaceutical fields [25]. In terms of sustainability, using ethanol as the only organic reagent for the extraction, isolation, and purification of the three carotenoids provides multiple advantages, including a green solvent, low production cost, and good product safety. Moreover, the use of ethanol also offers a significant advantage of low manufacturing cost, since the overwhelming majority of ethanol can be reused by several approaches in industrial production [26].

The study proposed a sustainable and efficient strategy for the integrated preparation of PER and DD-cycle carotenoids from wet *A. carterae*. This strategy possessed several advantages, including sustainability, green solvents, high efficiency, and multiple carotenoid products with high purity. Briefly, the approach is significant for the further valorization of *A. carterae* and the further application of PER and DD-cycle carotenoids.

## 2. Results and Discussion

### 2.1. Pigment Contents in Wet Amphidinium carterae Biomass

The results showed that the four compounds, such as chlorophyll a (Chl a), PER, DDX, and DTX, were the major pigments in wet *A. carterae* biomass. Detected pigments were quantified using their corresponding standards. In the study, the contents of Chl a, PER, DDX, and DTX in wet microalgal biomass were 5.35 ± 0.12, 3.32 ± 0.10, 0.42 ± 0.06, and 0.24 ± 0.05 mg/g wet *A. carterae*, respectively. The results suggested that wet *A. carterae* was an excellent microalgal feedstock for the concurrent preparation of PER and DD-cycle carotenoids due to its obvious advantages. Firstly, PER is a characteristic carotenoid of dinoflagellates [27]. DD-cycle carotenoids (including DDX and DTX) have been found in various styles of microalgae, especially in diatoms and dinoflagellates [28]. To meet the demand of the expected goals of extraction, the high content of the target molecules in the raw feedstock is essential. Interestingly, the levels of PER, DDX, and DTX in wet *A. carterae* were high enough for their expected preparation. Moreover, *A. carterae* can be cultured efficiently in various modes on a large scale [17]. Hence, the wet *A. carterae* was a promising raw material for the concurrent preparation of PER, DDX, and DTX.

### 2.2. Effects of Different Factors on the Pigment Extraction from Wet Amphidinium carterae

#### 2.2.1. Effects of Various Types of Solvents on Pigments Yield

**Figure 1:**
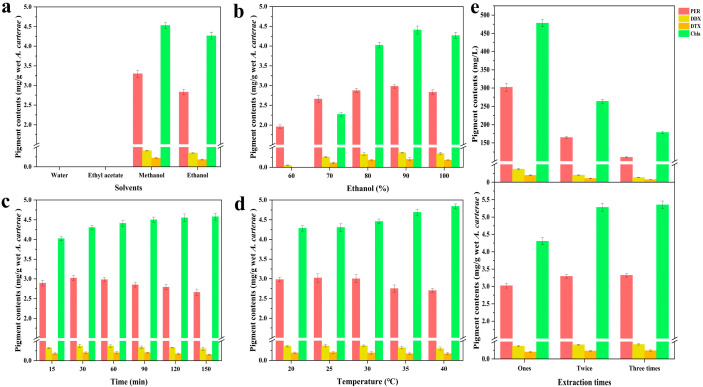
Influence of (**a**) extraction solvent type, (**b**) ethanol–water mixed solvent, (**c**) extraction duration, (**d**) temperature, and (**e**) the number of extraction times on pigment (PER, DDX, DTX, and Chl a) extraction from the microalga *A. carterae*. The error bars represent the SDs of three replicates.

In this study, the outcomes (Figure 1a) revealed that the four pigments (including Chl a, PER, DDX, and DTX) could not be effectively extracted by solvents such as water and ethyl acetate. The former result was primarily attributed to the inorganic characteristics of water, whereas the latter result was mainly due to the wet *A. carterae* (water content of the wet algal biomass: 91%, *w*/*w*), which could not achieve the effective dissolution of target carotenoids by ethyl acetate [29].

Notably, considering the PER yield, methanol demonstrated superior extraction ability compared to ethanol. The influence of these solvents on the pigments DDX, DTX, and Chl a was also examined, with the same rank: ethanol < methanol. These results could be explained by several factors. First, the content of Chl a was noticeably greater than that of the three carotenoids (PER, DDX, and DTX) in the wet microalgal biomass, resulting in its easier extraction from the wet feedstock. Second, the four pigments (PER, DDX, DTX, and Chl a) are organic polar compounds containing multiple polar functional groups [3,13,30]. The extraction efficiency of the four pigments was considerably affected by ethanol and methanol, with the relative polarity being 0.654 and 0.762, respectively. In terms of PER yield, better extraction efficiency could be achieved using more polar organic solvents (methanol), which is attributed to the similar dissolution mutual theory [8]. In terms of the three pigment yields (Chl a, DDX, and DTX), similar phenomena could also be observed in this study. Methanol demonstrated superior efficacy as a solvent for pigment extraction, attributable to its enhanced extraction capabilities. However, ethanol was ultimately determined to be the optimal solvent, owing to its several advantages, including safety, environmental sustainability, and cost-effectiveness [25].

#### 2.2.2. Effects of Different Ethanol Concentrations on Pigment Yield

The outcomes (Figure 1b) revealed that the PER yield increased remarkably with the ethanol concentration ranging from 60% to 90% ethanol (*v*/*v*), highlighting the significance of ethanol in extraction efficiency. The PER yield slightly declined with further increase in ethanol concentration from 90% to 100% ethanol (*v*/*v*). In terms of the yield of the three pigments (DDX, DTX, and Chl a), the different ethanol concentrations revealed a similar tendency to that of PER. These results could be explained by several factors. First, based on the chemical structures of the target pigments and the similar dissolution mutual theory, when the ethanol concentration ranged from 60% to 90% (*v*/*v*), an increase in ethanol concentration facilitated the pigment extraction from the wet microalgal biomass. However, the slight decrease in PER yield resulting from 100% ethanol extraction was mainly attributed to the structural characteristics of peridinin-chlorophyll-a-protein (PCP) and the extracellular layer of the wet *A. carterae* [31]. The extracellular layer, composed of acid mucopolysaccharides, could precipitate upon exposure to high ethanol concentrations [32]. The precipitation of acid mucopolysaccharides can play an essential role in hindering the achievement of efficient extraction of PER from the wet *A. carterae*. Similarly, the yields of pigments (DDX, DTX, and Chl a) smoothly reduced when ethanol concentration was more than 90%. Hence, 90% ethanol was the most suitable solvent for the extraction of PER, DDX, and DTX.

#### 2.2.3. Effects of Different Durations on Pigment Yield

The outcomes (Figure 1c) implied that Chl a yield showed a gradual increase with prolonged extraction time. However, PER yield smoothly ascended within the extraction time of 30 min, whereas it gradually descended when the duration exceeded 30 min. The extraction efficiency of DD-cycle carotenoids (DDX and DTX) showed similar trends in this study. These results could be explained by the following factors. Generally, prolonged exposure to air (oxygen) can lead to the degradation of carotenoids, including PER, DDX, and DTX [8]. Thus, 30 min was selected as the best extraction condition due to its high efficiency.

#### 2.2.4. Effects of Different Temperatures on Pigment Yield

The outcomes (Figure 1d) displayed that Chl a yield smoothly increased with the extraction temperatures within the temperature range between 20 and 40 °C. There was no significant difference in PER yield with temperatures ranging from 20 °C to 30 °C. However, as the temperature exceeded 30 °C, the PER yield gradually decreased. The extraction efficiency of the two carotenoids, DDX and DTX, showed similar trends in this study. The thermal instability of carotenoids is the main reason for these outcomes [21]. Therefore, 25 °C was the optimal condition due to its benefits in energy saving.

#### 2.2.5. Effects of Different Extraction Times on Pigment Yield

The results (Figure 1e) suggested that the yield of the four pigments improved as the extraction times increased, whereas the concentration of these pigments sharply decreased with the increasing number of extraction times. Remarkably, in one extraction process, the recovery rates (R_Ex_) of PER, DDX, and DTX exceeded 83%. The concentrations of PER, DDX, and DTX in *A. carterae* solution were approximately 302, 37, and 20 mg/L, respectively. Notably, the desired high enough concentration of PER and DD-cycle carotenoids obtained from a one-time extraction is vital for facilitating further separation procedures. Therefore, one-time extraction was the optimal condition for the concurrent extraction of PER and DD-cycle carotenoids.

In the study, some key extraction factors, such as various types of solvent, water/ethanol mixtures, duration, temperature, and extraction round, on the yield of four pigments (such as Chl a, PER, and DD-cycle carotenoids) from the wet *A. carterae* biomass (water content of the wet algal biomass: 91% *w*/*w*) were investigated. The optimum extraction conditions were determined as follows: 90% ethanol, 25 °C, and 30 min, with a liquid-to-solid ratio of 10:1, and a one-time extraction process. Under the conditions, the yields of PER and Chl a were 3.02 ± 0.04 and 4.30 ± 0.04 mg/g wet sample, respectively. The yields of DDX and DTX were 370.0 ± 20.0 and 200.0 ± 10.0 μg/g wet sample, respectively. Moreover, the R_EX_ values of PER, DDX, DTX, and Chl a were 90.96 ± 1.00%, 88.10 ± 1.12%, 83.33 ± 0.97%, and 80.37 ± 1.02%, respectively. To ensure the reproducibility of experimental results, some necessary procedures, including the microalgae cultural conditions and the harvest of the microalgal wet biomass, could facilitate the production of multiple batches of *A. carterae* wet biomass with similar physicochemical properties, such as the contents of carotenoids (specifically PER and DD-cycle carotenoids) and moisture content.

Technology for extracting total carotenoids from lyophilized *A. carterae* has been reported. An extraction procedure comprises saponification and organic solvent extraction [16,18]. An extraction method contains steps including cell breakage, saponification, and organic solvent extraction [33]. Notably, PER and DD-cycle carotenoids were efficiently extracted from wet *A. carterae* using ethanol extraction without the additional cell breakage and saponification processes in this study.

### 2.3. Isolation of Peridinin and Diadinoxanthin Cycle Carotenoids by Octadecylsilyl Open Column Chromatography

**Figure 2:**
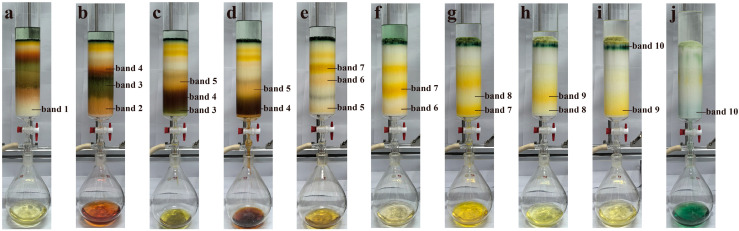
Elution process of the extraction solution from *A. carterae* with an ODS column chromatography. (**a**) elution using ethanol/water (65:35, *v*/*v*); (**b**,**c**) elution using ethanol/water (70:30, *v*/*v*); (**d**,**e**) elution using ethanol/water (75:25, *v*/*v*); (**f**–**h**) elution using ethanol/water (78:22, *v*/*v*); (**i**) elution using ethanol/water (80:20, *v*/*v*); (**j**) elution using absolute ethanol.

**Figure 3:**
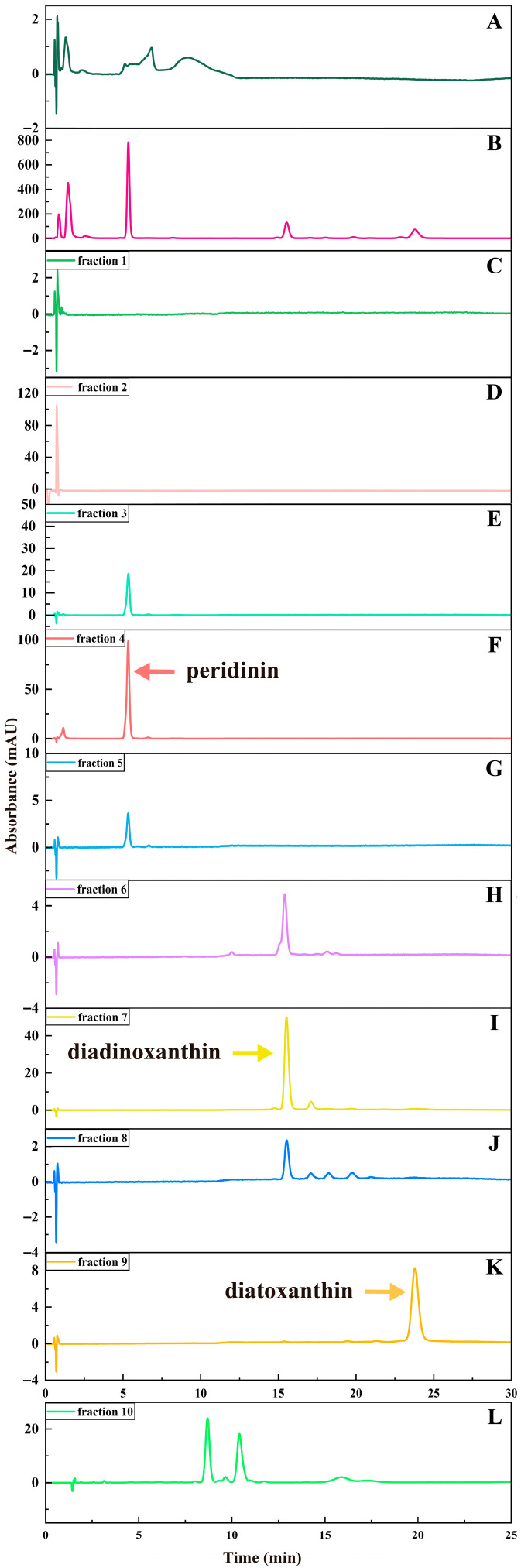
HPLC chromatograms of the extract of *A. carterae* and the fractions 1~10 obtained by ODS chromatography. (**A**): the extract of *A. carterae* (665 nm); (**B**): the extract of *A. carterae* (450 nm); (**C**–**K**): the fractions 1~9 (450 nm); (**L**): the fraction 10 (430 nm).

**Figure 4:**
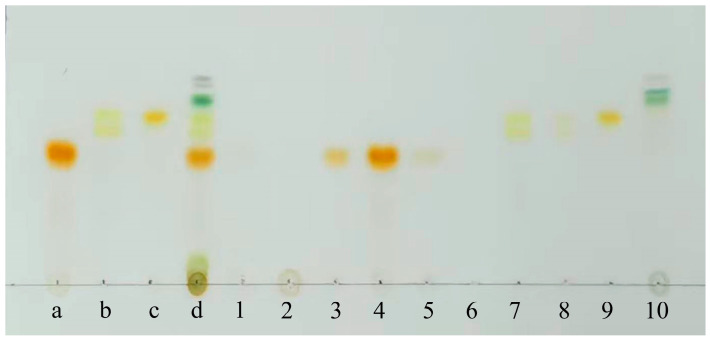
TLC analysis results: Sample a, PER standard; Sample b, DDX standard; Sample c, DTX standard; Sample d, *A. carterae* extraction solution; Samples 1~10, the eluted fractions corresponding to the bands 1~10 using an ODS column chromatography.

**Figure 5:**
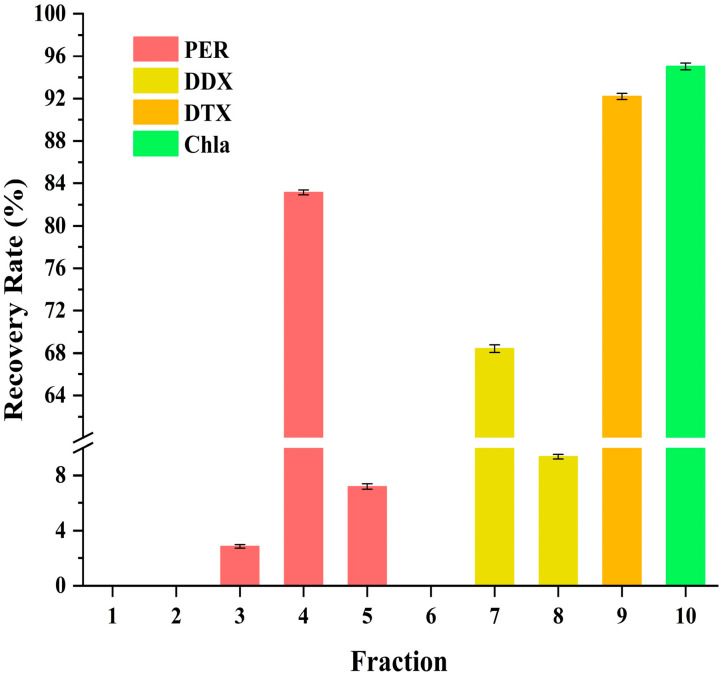
R_S_ values of pigments (PER, DDX, DTX, and Chl a) in the fractions 1~10.

In the experiment, the isolation of PER and DD-cycle carotenoids was successfully achieved using ODS open-column chromatography. Around 224 mL of *A. carterae* solution was obtained according to the optimum extraction conditions developed in this study. The concentration of PER, DDX, DTX, and Chl a was approximately 311, 38, 20, and 443 mg/L, respectively. To attain the required ethanol concentration (65%, *v*/*v*) for further ODS open-column chromatography, about 87 mL of water was introduced to the solution. Subsequently, the resulting solution was loaded on an ODS column and gradient eluted using a variety of mixtures with different ethanol and water ratios (65:35, 70:30, 75:25, 78:22, 80:20, 100:0, *v*/*v*). The separation procedure produced ten fractions according to the bands 1~10, each with its distinguishable color in the study. Figure 2, Figure 3, Figure 4 and Figure 5 exhibited the separation process as well as essential parameters related to the ODS open-column chromatography.

The outcomes showed that the polar impurities, which appeared green and red, were effectively removed by applying 65% and 70% ethanol, respectively (Figure 2, Figure 3 and Figure 4). Fraction 3, containing a small amount of PER, was eluted by 70% ethanol, with the recovery rate (R_S_) for PER of around 2.87% (Figure 5). Significantly, a black PER-rich fraction (PER concentration around 145.00 mg/L) was effectively isolated by 75% ethanol, achieving an R_S_ value for PER of approximately 83.14 ± 0.23% (Figure 5). Subsequently, the red fraction containing a small amount of PER was eluted by 75% ethanol, with the R_S_ value for PER of around 7.20% (Figure 5). Importantly, a golden DDX-rich fraction (DDX concentration around 20.17 mg/L) was effectively isolated by 78% ethanol, with the R_S_ value for DDX reaching 68.42 ± 0.36% (Figure 5). Afterward, a light yellow fraction that included DDX and various impurities was obtained using 78% ethanol. Notably, a yellow DTX-rich fraction (DTX concentration about 13.31 mg/L) was successfully obtained using 80% ethanol, with the R_S_ value for DTX reaching around 92.21 ± 0.29% (Figure 5). Ultimately, a green fraction primarily composed of non-polar impurities and Chl a was acquired using absolute ethanol, with the R_S_ value for Chl a reaching approximately 95.02 ± 0.32% (Figure 5). These experimental results were determined using HPLC and thin-layer chromatography (TLC) analysis (Figure 3 and Figure 4), confirming the successful separation of the target carotenoids (PER and DD-cycle carotenoids) by the ODS open-column chromatography in this experiment.

Notably, during the ODS open-column chromatography, bands 3~9 were observed, highlighting the co-extraction of various carotenoids in the *A. carterae* extraction solution. Generally, except for the target carotenoids, the simultaneous existence of different compounds in the wet *A. carterae* could pose significant challenges to further effective separation of target molecules. In this study, fractions 1~10 were effectively isolated based on their polarity differences according to the ODS chromatography separation principle. Interestingly, the close spacing between bands 3~9 highlighted a high degree of similarity in molecular polarity within these bands, which presented enormous challenges for efficient separation.

Several chromatographic methods have been developed for the isolation of PER from microalgal biomass, including HPLC and Chromatotron [11,12]. Additionally, several methods, such as HPLC and SGCC, have been established for the isolation of DDX and DTX [13]. In this study, ODS open-column chromatography resulted in three particularly noteworthy fractions, such as the PER-rich fraction, DDX-rich fraction, and DTX-rich fraction. Additionally, none of them contained Chl a (Figure 3). The experiment results showed that ODS chromatography was a successful and efficient method for the concurrent separation of the three target carotenoids from the wet *A. carterae*. Moreover, the three noteworthy fractions could create a solid and reliable foundation for the further purification of PER, DDX, and DTX.

Interestingly, the R_S_ value of PER in the PER-rich fraction (fraction 4) was around 83%, implying the possibility of other PER contained in other fractions. It was confirmed by the detection of PER in fractions 3~5 in this study (Figure 3). Similarly, DDX could be found in fractions 7 and 8 (Figure 3). Additionally, the R_S_ value of DDX in the DDX-rich fraction (fraction 7) was around 68%. These results suggested that most of each target carotenoid could be collected in a suitable fraction, wherein the concentration of the target carotenoid was high enough to facilitate further precipitation of the target carotenoid. Other fractions containing the target carotenoid had been discarded due to the low concentration of the target carotenoid or the coexistence of other impurities.

The concurrent isolation of PER, DDX, and DTX from microalgae still faces challenges due to some complex factors, including the coexistence of multiple components, similar polarity, chemical instability, and low content in the microalgal biomass. In this study, the effective isolation of the significant fractions—the PER-rich fraction, the DDX-rich fraction, and the DTX-rich fraction—demonstrated that ODS chromatography was an efficient and feasible method. Regarding cost-efficiency and sustainability, ODS column chromatography possesses significant advantages of the multiple recycling of ODS particles and ethanol. These sustainability features of ODS column chromatography could provide the feasibility of the technology on a large scale. Notably, the effective implementation of industrial ODS chromatography in the commercial production of docosahexaenoic acid ethyl ester can offer valuable insights and strategies for the future commercial integrated preparation of PER and DD-cycle carotenoids from the microalga *A. carterae* [24].

### 2.4. Purification of Peridinin and Diadinoxanthin Cycle Carotenoids Using Ethanol Precipitation

In this study, high-purity PER was obtained by ethanol precipitation. The influences of various ethanol concentrations on the PER precipitation were investigated. In the same way, high-purity DDX and DTX were also obtained by ethanol precipitation. The optimal ethanol precipitations of DDX and DTX were also investigated.

#### 2.4.1. Influences of Various Ethanol Concentrations in Peridinin Precipitation

**Figure 6:**
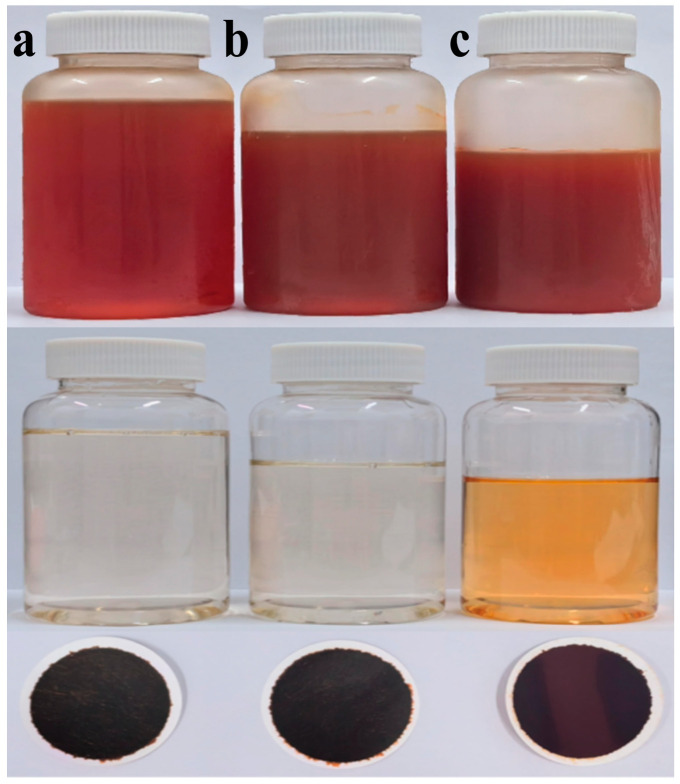
PER precipitation process from the wet *A. carterae*. PER precipitation after storage at −20 °C for 48 h, supernatants, and precipitants obtained by filtration through 0.45 μm membranes (from top to bottom). (**a**–**c**) PER precipitation in groups (**a**–**c**) was obtained from precipitation solutions with different concentrations of ethanol (from left to right: 30%, 35%, and 40% ethanol, respectively).

**Figure 7:**
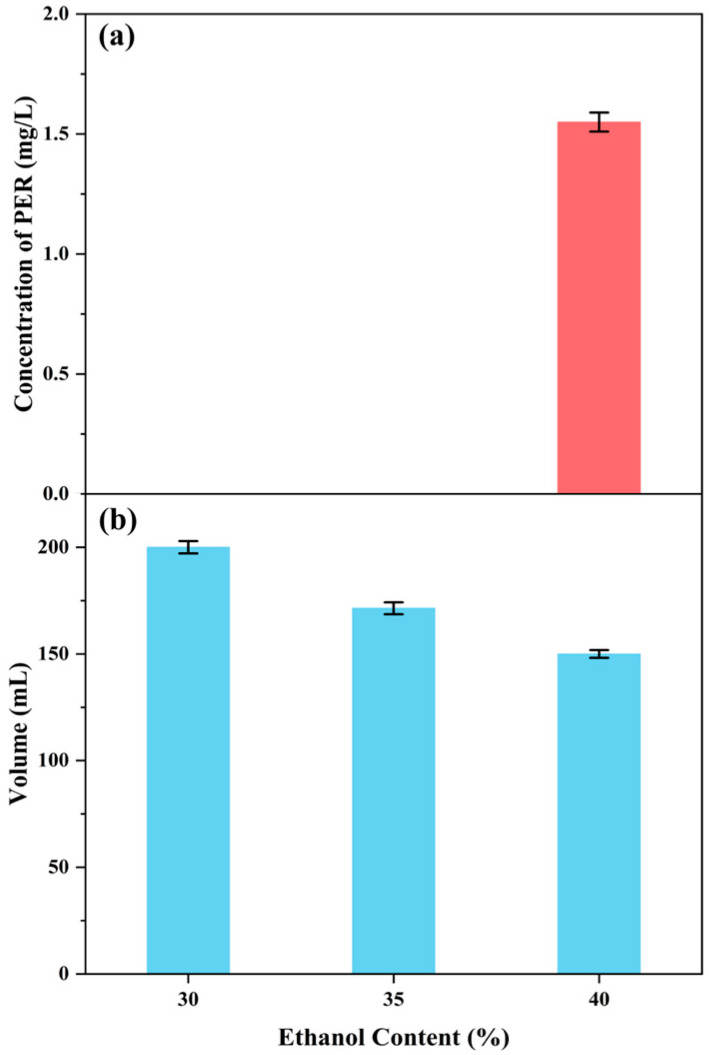
Influence of ethanol concentration (30~40%) on PER precipitation after storage at −20 °C for 48 h; (**a**) the concentration of PER in the supernatant; (**b**) the volume of the precipitation solution.

**Figure 8:**
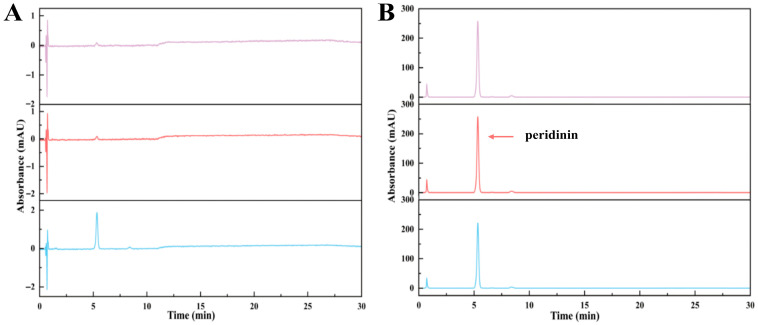
HPLC chromatograms of (**A**) supernatant with different ethanol concentrations (from up to down: 30%, 35%, and 40% ethanol, respectively); (**B**) purified PER obtained from precipitation solutions with different concentrations of ethanol (from up to down: 30%, 35%, and 40% ethanol, respectively).

**Figure 9:**
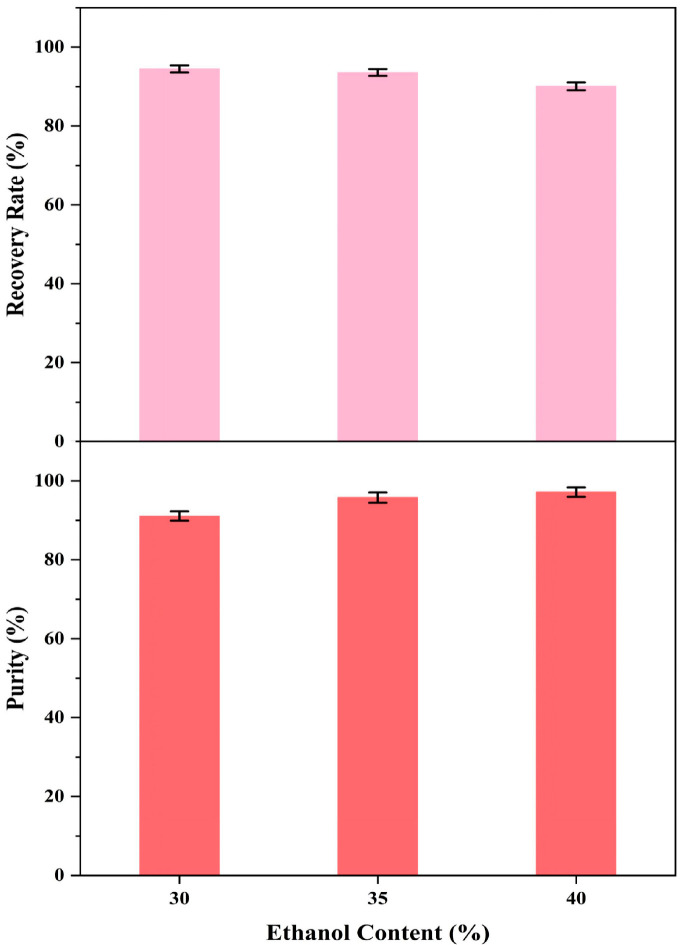
The R_P_ values and purity of PER.

High-purity PER was successfully gained by accurately adjusting the ethanol concentration in the precipitation systems in this study. Briefly, to achieve the desired ethanol concentrations in the precipitation systems, different amounts of water were introduced to the PER-rich fraction (PER concentration was about 145.00 mg/L) and then stored for 48 h at −20 °C under dim conditions to achieve the maximum PER precipitation. Subsequently, the purity and yield of PER precipitated at various ethanol concentrations (30%, 35%, and 40%, respectively) were evaluated. The related materials of PER precipitation, such as the related precipitation images, HPLC chromatograms, and essential parameters, were displayed (Figure 6, Figure 7, Figure 8 and Figure 9).

This study observed PER precipitation under the precipitation conditions with the three ethanol concentrations. The concentrations of PER in the supernatants containing 30% and 35% ethanol were not detected, whereas that in the supernatant with 40% ethanol was 1.55 mg/L (Figure 7 and Figure 8). The recovery rate (R_P_) for the obtained solid PER decreased with the increase in ethanol concentration. In the precipitation systems containing 30% and 35% ethanol, the R_P_ values of PER were more than 93%, whereas that was measured at about 90% under the system containing 40% ethanol (Figure 9). These results emphasized that appropriate ethanol concentration had a significant impact on the recovery of high-purity products during the PER precipitation procedure. These outcomes were mainly attributed to the following reasons. Air and heat exposure could cause PER to degrade [21]. The ethanol concentration in the precipitation system generates a significant impact on the solubility of the target molecule [25]. The R_P_ value of solid PER in the system containing 40% ethanol was mainly attributed to the loss of a minor amount of PER dissolved in the supernatant.

Interestingly, the purity of the obtained solid PER increased with the ethanol concentration ranging from 30% to 40% in the study. The purities of the obtained PER were more than 95% in the supernatants containing 35% ethanol and 40% ethanol, whereas that of the obtained PER was about 91% in the supernatant containing 30% ethanol (Figure 9). These outcomes were mainly attributed to the solubility of the lipid compounds, which increased with higher ethanol concentrations [34]. The dissolution of lipid impurities in precipitation systems with 35% and 40% ethanol effectively improved the purity of PER. Hence, regarding the recovery rate and purity of the final product, 35% ethanol was selected as the best precipitation condition for PER precipitation. The purity of the solid PER was around 95%, and the R_P_ value of PER was approximately 93%.

#### 2.4.2. Influences of Various Ethanol Concentrations on Diadinoxanthin Precipitation

**Figure 10:**
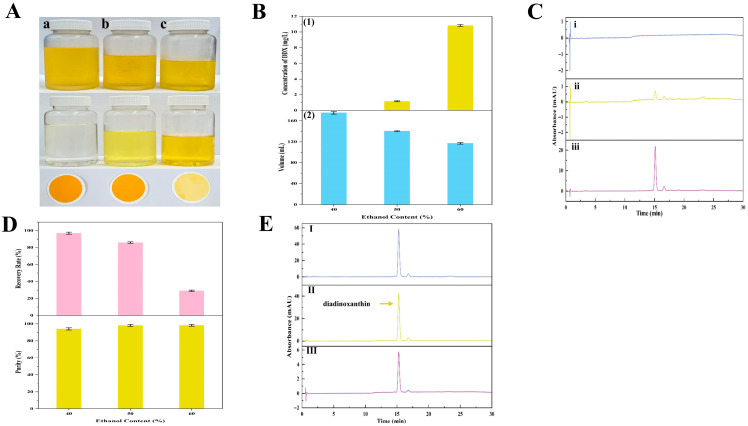
The precipitation process and key parameters of DDX precipitation. (**A**) Images of the DDX precipitation process from the wet *A. carterae*. DDX precipitation after storage at −20 °C for 48 h, supernatants, and precipitants obtained by filtration through 0.45 μm membranes (from top to bottom). (a~c) DDX precipitation in groups (a~c) was obtained from precipitation solutions with different concentrations of ethanol (from left to right: 40%, 50%, and 60% ethanol, respectively); (**B**) Influence of ethanol concentration (40~60%) on DDX precipitation after storage at −20 °C for 48 h; (1) the concentration of DDX in the supernatant; (2) the volume of the precipitation solution; (**C**) HPLC chromatograms of supernatant with different ethanol concentrations (from up to down (i–iii): 40%, 50%, and 60% ethanol, respectively); (**D**) the R_P_ values and purity of DDX; and (**E**) HPLC chromatograms of purified DDX obtained from precipitation solutions with different concentrations of ethanol (from up to down (I–III): 40%, 50%, and 60% ethanol, respectively).

In a similar pattern, DDX was successfully purified by changing the ethanol concentration in the precipitation systems. Key materials concerning DDX precipitation, such as the precipitation images, HPLC chromatograms, and critical parameters, were exhibited (Figure 10). The results demonstrated that under the precipitation systems with ethanol concentrations of 40%, 50%, and 60%, the concentrations of DDX in the supernatants were 0, 1.17, and 10.83 mg/L, respectively (Figure 10B,C). Moreover, the R_P_ values of the corresponding solid DDX were about 96%, 85%, and 29%, respectively (Figure 10D). The purities of the corresponding DDX were approximately 94%, 98%, and 98%, respectively (Figure 10D). These outcomes revealed that a suitable ethanol concentration was significant for keeping a high recovery rate of DDX during ethanol precipitation. The results were primarily due to the loss of DDX dissolved in the supernatant with excessive ethanol concentration (60% ethanol). Therefore, 50% ethanol was identified as the best condition for DDX precipitation. The purity of the solid DDX was around 98%, and the R_P_ value of DDX was around 85%. The optimal condition of DDX precipitation obtained in this study was consistent with the former report [8].

#### 2.4.3. Influences of Various Ethanol Concentrations in Diatoxanthin Precipitation

**Figure 11:**
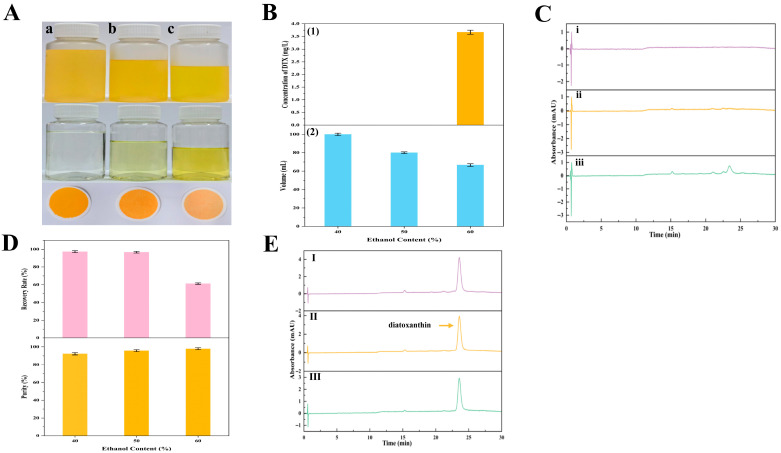
The precipitation process and key parameters of DTX precipitation. (**A**) Images of the DTX precipitation process from the wet *A. carterae*. DTX precipitation after storage at −20 °C for 48 h, supernatants, and precipitants obtained by filtration through 0.45 μm membranes (from top to bottom). (a~c) DTX precipitation in groups (a~c) was obtained from precipitation solutions with different concentrations of ethanol (from left to right: 40%, 50%, and 60% ethanol, respectively); (**B**) Influence of ethanol concentrations (40~60%) on DTX precipitation after storage at −20 °C for 48 h; (1) The concentration of DTX in the supernatant; (2) The volume of the precipitation solution; (**C**) HPLC chromatograms of supernatant with different ethanol concentrations (from up to down (i–iii): 40%, 50%, and 60% ethanol, respectively); (**D**) The R_P_ value and purity of DTX; and (**E**) HPLC chromatogram of purified DTX obtained from precipitation solutions with different concentrations of ethanol (from up to down (I–III): 40%, 50%, and 60% ethanol, respectively).

Similarly, DTX with high purity was successfully produced using an ethanol precipitation process similar to the procedure described above in this study. Key materials concerning DTX precipitation, including the precipitation images, HPLC chromatograms, and important parameters, were shown (Figure 11). The results revealed that in the precipitation systems containing 40%, 50%, and 60% ethanol, the R_P_ values of solid DTX were around 97%, 96%, and 61%, respectively (Figure 11D). The purities of the corresponding DTX were approximately 92%, 95%, and 98%, respectively (Figure 11D). Hence, 50% ethanol was identified as the best precipitation condition based on its high recovery rate and high purity of solid DTX. The purity of solid DTX was around 95%, and the R_P_ value of DTX was about 96%.

Ethanol precipitation is a widely utilized purification technique for various chemicals due to its remarkable advantages of simple operation, low cost, sustainability, and simplicity in industrial-scale production [35]. Three carotenoid compounds, including PER, DDX, and DTX, were purified from the wet *A. carterae* using ethanol precipitation for the first time in this study. The results strengthened the fact that ethanol precipitation under low temperature (−20 °C) was an efficient purification method for various xanthophyll compounds [8,21]. Generally, xanthophyll compounds possess chemical instability when exposed to heat conditions. Appropriate ethanol concentration during the precipitation process was essential for the balance of the high recovery rate and high purity of the target xanthophyll compound. For the PER precipitation, the optimum conditions were 35% ethanol, −20 °C, and 48 h. However, the purification of DDX and DTX shared the same precipitation conditions: 50% ethanol, −20 °C, and 48 h. In summary, the study introduced a sustainable and effective strategy for the co-production of high-purity PER and DD-cycle carotenoids from the wet *A. carterae*. Under the optimal processing conditions, the purities of solid PER, DDX, and DTX were around 95%, 98%, and 95%, respectively. The overall recovery rates (R_T_) of PER, DDX, and DTX were around 70%, 51%, and 74%, respectively.

### 2.5. Identification of Peridinin and Diadinoxanthin Cycle Carotenoids from Amphidinium carterae

The purified product was determined as PER according to the fragment pattern at *m*/*z* 631.3627 and 613.3512, which corresponded to [M + H]^+^ and [M − H_2_O + H]^+^ (Figure S1). In a similar pattern, the determination of the purified product as DDX was established by examining the fragment pattern of *m*/*z* 583.4131, which corresponded to [M + H]^+^ (Figure S2). Moreover, the purified product was identified as DTX by evaluating the fragment pattern of *m*/*z* 567.4174, which corresponded to [M + H]^+^ (Figure S3).

The NMR spectra provide a robust technique for elucidating the chemical structures of the purified PER, DDX, and DTX. Table S1 and Table S2 offer detailed attribution and structural analysis data for the ^1^H and ^13^C spectra, respectively. Based on the data, the purified compounds were determined to be all-*trans* PER, all-*trans* DDX, and all-*trans* DTX based on existing studies [36,37,38]. The structures and NMR spectra of the purified PER, DDX, and DTX are listed in Figure S4, Figure S5, Figure S6, Figure S7, Figure S8, Figure S9 and Figure S10. HPLC chromatograms of the purified all-*trans* PER, all-*trans* DDX, and all-*trans* DTX are presented in Figure S11, Figure S12 and Figure S13. In addition, their absorption spectra recorded during HPLC-DAD analysis are shown in Figure S14, Figure S15, Figure S16. Light-absorption properties provide one of the essential criteria for the characterization of carotenoids [39]. The spectroscopic observations in this study were consistent with those reported earlier [40,41].

### 2.6. Antioxidant Activity of Peridinin and Diadinoxanthin Cycle Carotenoids

**Figure 12:**
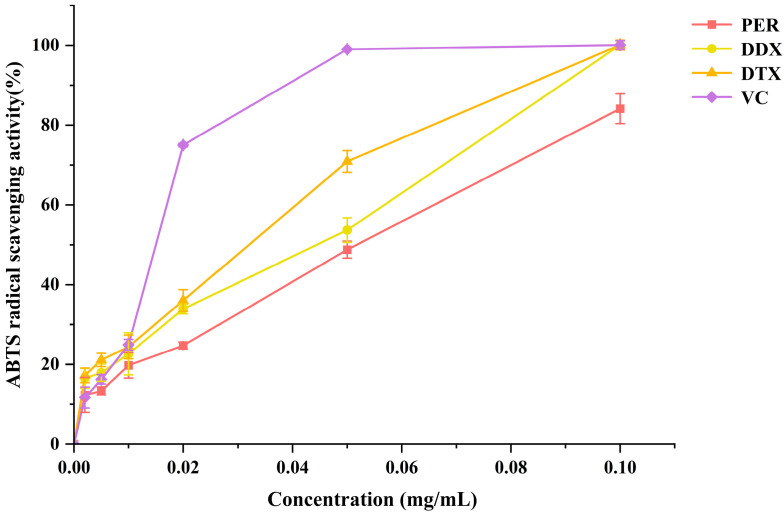
The free radical scavenging activities of purified PER, DDX, and DTX from *A. carterae* at various concentrations were assessed using the ABTS assay. Ascorbic acid was used as the reference standard, and each experiment was performed in triplicate.

Carotenoids are a class of antioxidant compounds that have the potential to exert beneficial effects on human health [42]. To evaluate the antioxidant capacity of PER, DDX, and DTX purified from *A. carterae*, the ABTS radical scavenging assay was performed. Ascorbic acid was used as a standard antioxidant and positive control. The scavenging effects of purified PER, DDX, and DTX on ABTS radicals are presented in Figure 12. The EC_50_ values of purified PER, DDX, and DTX were determined to be 0.052, 0.044, and 0.032 mg/mL, respectively. These results indicated that DTX exhibited a higher scavenging ability for ABTS^+^ radicals compared to DDX and PER. This enhanced activity could likely be attributed to the longer conjugated double bonds in the structure of DTX, as well as the absence of epoxy groups. Collectively, these structural features suggested that DTX possessed superior antioxidant capacity.

The biomass of *A. carterae* comprises various carotenoids [19]. The concurrent preparation of feasible carotenoids from the wet *A. carterae* depends on several factors, including high content of target carotenoids in the microalgal biomass, appropriate polarity of the carotenoids, and the effective separation of these compounds under the specified extraction conditions [8]. Presently, no report on the concurrent preparation of PER, DDX, and DTX from microalgal biomass has been discovered. Taken together, a novel strategy for the integrated preparation of PER and DD-cycle carotenoids from the wet *A. carterae* was developed for the first time in the study. Multiple high-purity carotenoids, including PER, DDX, and DTX, were successfully obtained.

## 3. Materials and Methods

### 3.1. Chemicals

PER (purity > 95%) was purchased from ChromaDex, Los Angeles, CA, USA. Chl a, DDX, and DTX (purity > 95%) were provided by Sigma Aldrich, Shanghai, China. Acetonitrile (HPLC grade) and methanol (HPLC grade) were sourced from Anpel, Shanghai, China. Reagents of analytical grade were obtained from Sinopharm, Shanghai, China.

### 3.2. Microalgae Cultural Conditions

The *A. carterae* strain (HL-012) was supplied by the State Key Laboratory of Bioreactor Engineering, East China University of Science and Technology. Cultures were initially maintained in 250 mL shake flasks using 100 mL autoclaved KWF seawater culture medium with the initial inoculation density of 1.5 × 10^5^ cells/mL [43]. The light intensity was 55 μmol/m^2^/s, the shaker’s speed was 120 rpm, and the temperature was maintained at 25 °C. When microalgal cells reached the early logarithmic stage of growth (about one week), they were inoculated into bubble-column photobioreactors with an initial inoculation density of 1.5 × 10^5^ cells/mL. The culture volume of the second stage was 500 mL, and the photobioreactors were placed in a lighting incubator with a light intensity of 55 μmol/m^2^/s and a temperature of 25 °C, as well as continuous admission of sterile air. About two weeks after microalgal cells attained the late logarithmic stage of growth, the yield of PER, DDX, and DTX reached 32.44, 4.12, and 2.38 mg/L in the conical flask system, respectively. Then the microalgal biomass was collected via centrifugation at 8000 rpm for 5 min. The resulting wet biomass (water content of the wet algal biomass: 91%, *w*/*w*) was stored at −20 °C for further procedure. These procedures facilitated the production of multiple batches of *A. carterae* wet biomass with similar physicochemical properties, including the contents of carotenoids (specifically PER and DD-cycle carotenoids) and moisture content, thereby ensuring the reproducibility of experimental results.

### 3.3. Quantification of Pigments in Amphidinium carterae

Pigment quantification was conducted using a previously reported approach with slight modifications [8]. Briefly, wet *A. carterae* biomass (0.1 g) was adequately extracted with 40 mL of methanol for 2 h under ice bath conditions. The obtained extraction solution was filtered through a 0.22-μm membrane for further HPLC analysis. To avoid pigment degradation, the extraction process was conducted in the dark.

Samples were analyzed using an Agilent 1260 series HPLC system. HPLC analysis of three carotenoids (PER, DDX, and DTX) was performed using a C18 column (Agilent XDB Eclipse, 150 mm × 4.6 mm, 5 μm particle size), equipped with a diode array detector. The samples were gradient-eluted using acetonitrile (solvent A) and water (solvent B) as gradient elution solvents. The gradient elution was programmed as follows: 0–10 min, 73% A; 10–11 min, 73–80% A; 11–27 min, 80% A; 27–30 min, 80–73% A. The column temperature was controlled at 25 °C, with an injection volume of 10 µL. The flow rate was 2.0 mL/min, and detection was at 450 nm (Figure S17).

Quantification analysis of Chl a was conducted using a C18 column (YMC Pack ODS-A, 4.6 mm × 250 mm, 5 µm particle size) controlled at 25 °C. An isocratic elution was performed utilizing 100% methanol as the mobile phase, with a flow rate maintained at 2 mL min^−1^. The injection volume was 10 µL, and the detection was at 430 nm (Figure S17).

### 3.4. Optimization of Peridinin and Diadinoxanthin Cycle Carotenoids Extraction from Amphidinium carterae

The influences of several key factors on the extraction of four pigments, such as PER, DDX, DTX, and Chl a, from the wet biomass of *A. carterae* were investigated in the study. The one-variable-at-a-time approach was applied to optimize the process [44]. The wet microalgal biomass (about 0.5 g) was extracted using 5.0 mL of solvent with a shaking speed of 150 rpm for 1 h at 25 °C in darkness. The effects of different factors were assessed, including different types of solvent (water, ethyl acetate, ethanol, and methanol), different water/ethanol mixtures (*v*/*v*) (from 60% ethanol to 100% ethanol), extraction duration (from 15 to 150 min), temperature (from 20 °C to 40 °C), and extraction times (once, twice, or three times), on the yields of the four pigments from the wet microalgal biomass. The resulting extract was centrifuged at 8000 rpm for 15 min at 4 °C, and filtered through a 0.22-μm membrane for further HPLC analysis. The whole extraction process was conducted in darkness, and each experiment was independently repeated three times.

The concentrations of the four pigments (PER, DDX, DTX, and Chl a) present in the extract solution (P_EX_) were quantified in milligrams per gram of the initial wet microalgal biomass used for extraction (W_0_), as specified in Equation (1).

P_EX_ = (C_EX_ × V_EX_)/W_0_ (1)

where C_EX_ denotes the quantified concentration of pigments within the extraction solution (mg/mL), and V_EX_ refers to the volume of the solution (mL). Regarding the extraction process, the recovery rate of the pigments (R_EX_) was determined using Equation (2).

R_EX_ (%) = (M_EX_/M_0_) × 100 (2)

where M_EX_ represents the mass of pigments present in the extraction solution (mg), and M_0_ signifies the mass of pigments contained within the wet microalgal *A. carterae* biomass (mg).

### 3.5. Concurrent Isolation of Peridinin and Diadinoxanthin Cycle Carotenoids Using Octadecylsilyl Column Chromatography

Wet *A. carterae* biomass (23.0 g) was extracted using the optimized conditions. A suitable amount of water was introduced to the *A. carterae* solution to adjust the ethanol concentration to 65% (*v*/*v*). The resulting mixture was then transferred into a column (280 mm ×76 mm i.d.) pre-packed with a sufficient quantity of ODS fillers (230 g dry weight, YMC^®^ GEL, ODS-A-HG, S-50 µm, YMC, Kyoto, Japan). Gradient elution was subsequently performed using various ethanol/water mixtures with a flow rate of 0.9 mL/s. Subsequently, the sample was first washed with 65% ethanol (*v*/*v*) and then sequentially with 70%, 75%, 78%, 80%, and 100% ethanol. The eluted fractions were collected based on their respective colors. Additionally, a total of ten fractions were gained with the approximate volumes as follows: 790, 410, 220, 400, 250, 380, 290, 570, 320, and 2000 mL, respectively. TLC and HPLC were used to evaluate the target pigments in each fraction.

The recovery rate of pigments (R_S_) during the concurrent isolation of PER, DDX, and DTX was determined using Equation (3).

R_S_ (%) = M_S_/M_EX_ × 100 (3)

where M_S_ represents the mass of the pigments in the respective fraction eluted through ODS chromatography (mg), and M_EX_ denotes the mass of the pigments present in the extract solution (mg).

### 3.6. Ethanol Precipitation for the Purification of Peridinin and Diadinoxanthin Cycle Carotenoids

#### 3.6.1. Optimization of Ethanol Concentration in Peridinin Precipitation

To determine the optimal condition for PER precipitation, 80 mL of PER-rich fractions (PER concentration was about 145.00 mg/L) were combined with different quantities of water to produce the desired ethanol concentrations (30%, 35%, and 40%, *v*/*v*) in the precipitation systems. The resulting mixtures were deposited in darkness at −20 °C for 48 h to facilitate PER precipitation, followed by filtration through a 0.45 μm membrane. The obtained black precipitates were then lyophilized and dissolved in methanol for further analysis.

#### 3.6.2. Optimization of Ethanol Concentration in Diadinoxanthin Precipitation

Similarly, the effects of precipitation systems with different ethanol concentrations (40%, 50%, and 60% *v*/*v*) on DDX precipitation were evaluated in the study. In brief, 90 mL of fractions enriched with DDX (DDX concentration approximately 20.17 mg/L) were combined with varying amounts of water. The following experimental process was carried out according to the earlier described PER precipitation.

#### 3.6.3. Optimization of Ethanol Concentration in Diatoxanthin Precipitation

In a similar pattern, the effects of different ethanol concentrations (40%, 50%, and 60% *v*/*v*) on DTX precipitation were studied. Briefly, 50 mL of DTX-rich fractions (DTX concentration around 13.31 mg/L) were combined with appropriate quantities of water, following the previously described PER precipitation method.

The recovery rate of ethanol precipitation (R_P_) of PER, DDX, and DTX was indicated using Equation (4).

R_P_ (%) = M_P_/M_S_ × 100 (4)

where M_P_ represents the mass of the carotenoids precipitated in ethanol solution (mg), and M_S_ denotes the mass of the carotenoids in the fractions derived by ODS column chromatography (mg).

The overall recovery rate (R_T_) of the pigments was indicated using Equation (5).

R_T_ (%) = R_EX_ × R_S_ × R_P_ × 100 (5)

where R_EX_, R_S,_ and R_P_ represent the pigment recoveries obtained from the three independent procedures of solvent extraction, ODS open-column chromatography, and ethanol precipitation, respectively.

### 3.7. Thin-Layer Chromatography Analysis of Pigments

The TLC method was employed to analyze pigments in fractions derived by ODS column chromatography. Briefly, the samples were spotted on a TLC plate precoated with silica gel HF_254_, which was subsequently positioned in a TLC developing chamber containing a solvent system composed of acetone and petroleum ether (boiling point range of 60 to 90 °C) in a 3:7 ratio as the developing agent. Following the development process, the plate was promptly photographed under indoor daylight conditions.

### 3.8. Characterization of Peridinin and Diadinoxanthin Cycle Carotenoids from Amphidinium carterae

The electrospray ionization mass spectrometry (ESI-MS) analysis was performed using an electrospray ionization-quadrupole-time-of-flight mass spectrometry instrument (ESI-Q-TOF MS; Waters, Milford, CT, USA). The ^1^H and ^13^C NMR experiments were carried out by a Bruker 600 MHz NMR system (Bruker Ascend 600 MHz, Baden-Württemberg, Germany, ^1^H at 600 MHz, ^13^C at 150 MHz).

### 3.9. Determination of Antioxidant of Peridinin and Diadinoxanthin Cycle Carotenoids

The antioxidant activities of PER, DDX, and DTX were evaluated using the modified ABTS method [45]. The ABTS free radical scavenging assay was slightly adapted by Osman et al. [46]. A pre-configured stock solution of ABTS radicals was prepared by mixing 7 mM ABTS diammonium salt with 2.45 mM potassium persulfate and allowing the reaction to proceed overnight in the dark. Prior to use, the stock solution was diluted with methanol to achieve an absorbance of 0.700 ± 0.020 at 734 nm. In brief, 100 μL of ethanolic solutions of PER, DDX, and DTX (concentrations ranging from 0.001 to 0.1 mg/mL) were mixed with the ABTS solution at a 1:3 volume ratio. The mixture was kept at 25 °C in the dark for 10 min. The absorbance was subsequently measured at 734 nm using a microplate reader. Ascorbic acid was used as a positive control. The scavenging ability was calculated according to the following formula: ABTS radical scavenging activity (%) = [A_Control_ − (A_Sample_ − A_SampleBlank_)/A_Control_] × 100, where A_Control_ represents the absorbance value of ABTS^+^ alone, A_SampleBlank_ corresponds to the absorbance of the sample without ABTS^+^, and A_Sample_ denotes the absorption value containing the sample and ABTS^+^.

### 3.10. Statistical Analysis

Each experiment was conducted in triplicate, with error bars indicating the standard deviation. Statistical analyses were performed using IBM SPSS Statistics version 26, and a significance level of *p* < 0.05 was considered statistically significant. The least significant difference (LSD) test was utilized to evaluate statistical comparisons.

## 4. Conclusions

This study introduced a pioneering strategy for the co-production of high-purity PER and DD-cycle carotenoids from the wet *A. carterae*. The methodology encompassed four principal processes: microalgal cultivation, ethanol extraction, ODS open-column chromatography, and ethanol precipitation. Under the optimized extraction and purification conditions in this study, it could be readily inferred that the mass of the three carotenoids, PER, DDX, and DTX, extracted from each kilogram of the wet microalgae biomass was approximately 2360, 218, and 178 mg, respectively. Additionally, the purities of all three carotenoids were greater than 95%. This research represented a significant advancement in the concurrent preparation of PER and DD-cycle carotenoids from microalgal biomass and emphasized *A. carterae* as a promising species for production. This biorefinery approach held substantial significance for the future application of PER and DD-cycle carotenoids, as well as for the integrated biorefinery potential of wet *A. carterae*.

## Data Availability

Data is contained within the article and Supplementary Materials.

## Supplementary Materials

The following supporting information can be downloaded at https://www.mdpi.com/article/10.3390/md23100405/s1: Figures S1–S3: HRMS spectra of the purified peridinin, diadinoxanthin, and diatoxanthin; Figures S4–S9: NMR spectra of the purified peridinin, diadinoxanthin, and diatoxanthin; Figure S10: Structures of the purified all-*trans* PER, all-*trans* DDX, and all-*trans* DTX isolated from *A. carterae*; Figures S11–S13: HPLC chromatograms (DAD, 450 nm) of the purified all-*trans* peridinin, all-*trans* diadinoxanthin, and all-*trans* diatoxanthin; Figures S14–S16: Absorption spectra of the purified all-*trans* peridinin, all-*trans* diadinoxanthin, and all-*trans* diatoxanthin recorded during HPLC-DAD analysis; Figure S17: HPLC chromatograms of four pigments standards; (**A**) individual peaks are 1. peridinin, 2. diadinoxanthin, 3. diatoxanthin; (**B**) individual peak is 4. Chlorophyll a. Tables S1 and S2: NMR data for purified peridinin, diadinoxanthin, and diatoxanthin.
