# Supplementary material for: Valorization of Amphidinium carterae for Integrated Preparation of Peridinin and Diadinoxanthin Cycle Carotenoids
Source: Mar Drugs. 2025 Oct 17;23(10):405. doi: 10.3390/md23100405 (PMC12565439; doi:10.3390/md23100405)
Supplement: Supplementary file 1 [file marinedrugs-23-00405-s001.zip › marinedrugs-3899144-supplementary.pdf]

Supplementary Material

Valorization of *Amphidinium carterae* for integrated preparation of peridinin and diadinoxanthin cycle carotenoids

Yi Li [1,†], GengJie Zhuang [2,†], Xuan Zhang [1], Wei Cui [3], ZhiWei Hong [4,5], JianHua Fan [4,5], JinRong Zhang [1,*], XiaoJun Yan[6,*]

1 College of Food Sciences and Engineering, Ningbo 315211, China; liyi20241205@163.com (Y.L.); 19857990154@163.com (X.Z.)
2 School of Marine Sciences, Ningbo University, Ningbo 315211, China; zhuanggengjie123@163.com (G.Z.)
3 Health Science Center, Ningbo University, Ningbo 315211, China; cuiwei@nbu.edu.cn (W.C.)
4 State Key Laboratory of Bioreactor Engineering, East China University of Science and Technology, Shanghai 200237, China; y30230492@mail.ecust.edu.cn (Z.H.); jhfan@ecust.edu.cn (J.F.)
5 Department of Applied Biology, East China University of Science and Technology, Shanghai 200237, China; y30230492@mail.ecust.edu.cn (Z.H.); jhfan@ecust.edu.cn (J.F.)
6 Key Laboratory of Applied Marine Biotechnology of Ministry of Education, Ningbo University, Ningbo 315211, China
* Correspondence: zhangjinrong@nbu.edu.cn (J.Z.); yanxiaojun@nbu.edu.cn (X.Y.); Tel./Fax: +86-574-8760-0458 (J.Z.); +86-574-8760-0556 (X.Y.)
† These authors contributed equally to this work.

Table of Contents

Figure S1. HRMS spectrum of the purified peridinin (in CH_3OH).
Figure S2. HRMS spectrum of the purified diadinoxanthin (in CH_3OH).
Figure S3. HRMS spectrum of the purified diatoxanthin (in CH_3OH).
Figure S4. ^{1}H NMR spectrum (600 MHz) of the purified peridinin (in $CDCl_3$).
Figure S5. ^{13}C NMR spectrum (150 MHz) of the purified peridinin (in $CDCl_3$).
Figure S6. ^{1}H NMR spectrum (600 MHz) of the purified diadinoxanthin (in CD_3COCD_3)
Figure S7. ^{13}C NMR spectrum (150 MHz) of the purified diadinoxanthin (in CD_3COCD_3).
Figure S8. ^{1}H NMR spectrum (600 MHz) of the purified diatoxanthin (in $CDCl_3$).
Figure S9. ^{13}C NMR spectrum (150 MHz) of the purified diatoxanthin (in $CDCl_3$).
Figure S10. Structures of the purified all-*trans* peridinin, all-*trans* diadinoxanthin, and all-*trans* diatoxanthin isolated from *Amphidinium carterae.*
Figure S11. HPLC chromatogram (DAD, 450 nm) of the purified all-*trans* peridinin.
Figure S12. HPLC chromatogram (DAD, 450 nm) of the purified all-*trans* diadinoxanthin.
Figure S13. HPLC chromatogram (DAD, 450 nm) of the purified all-*trans* diatoxanthin.
Figure S14. Absorption spectrum of the purified all-*trans* peridinin recorded during HPLC-DAD analysis.
Figure S15. Absorption spectrum of the purified all-*trans* diadinoxanthin recorded during HPLC-DAD analysis.
Figure S16. Absorption spectrum of the purified all-*trans* diatoxanthin recorded during HPLC-DAD analysis.
Figure S17. HPLC chromatograms of four pigments standards; (A) Individual peaks are 1. peridinin, 2. diadinoxanthin, 3. diatoxanthin; (B) Individual peak is 4. Chlorophyll a.
Table S1. ^{1}H NMR data for purified peridinin, diadinoxanthin, and diatoxanthin.
Table S2. ^{13}C NMR data for purified peridinin, diadinoxanthin, and diatoxanthin.

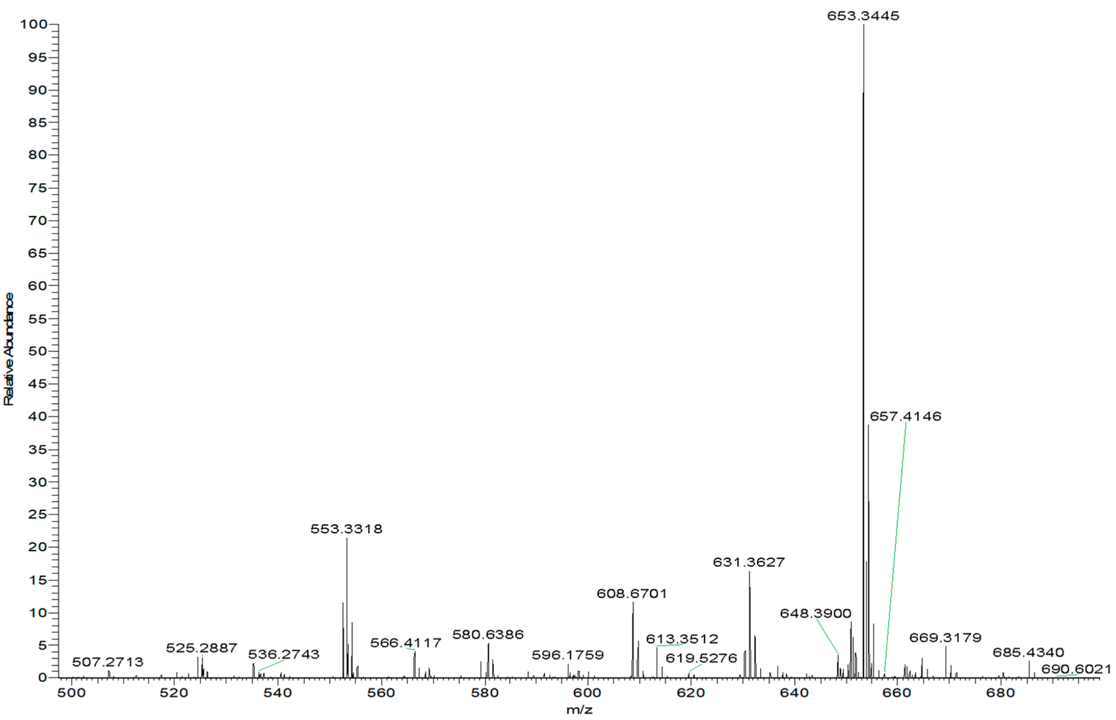

Figure S1. HRMS spectrum of the purified peridinin (in CH_3OH).

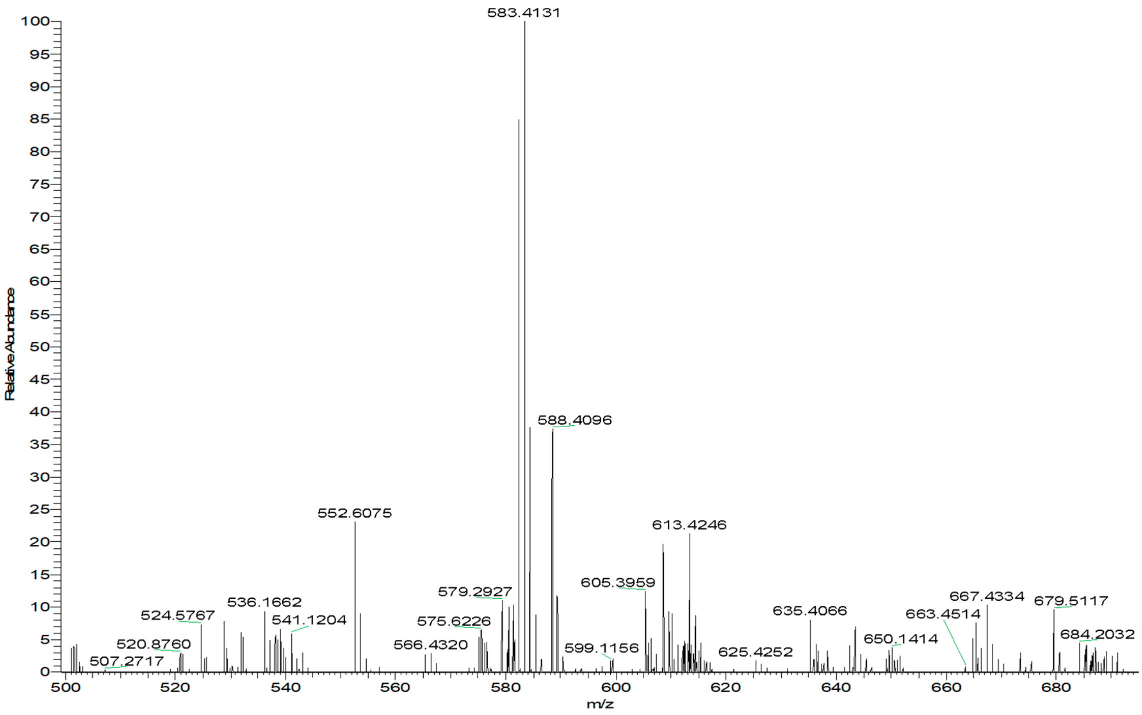

Figure S2. HRMS spectrum of the purified diadinoxanthin (in CH_3OH).

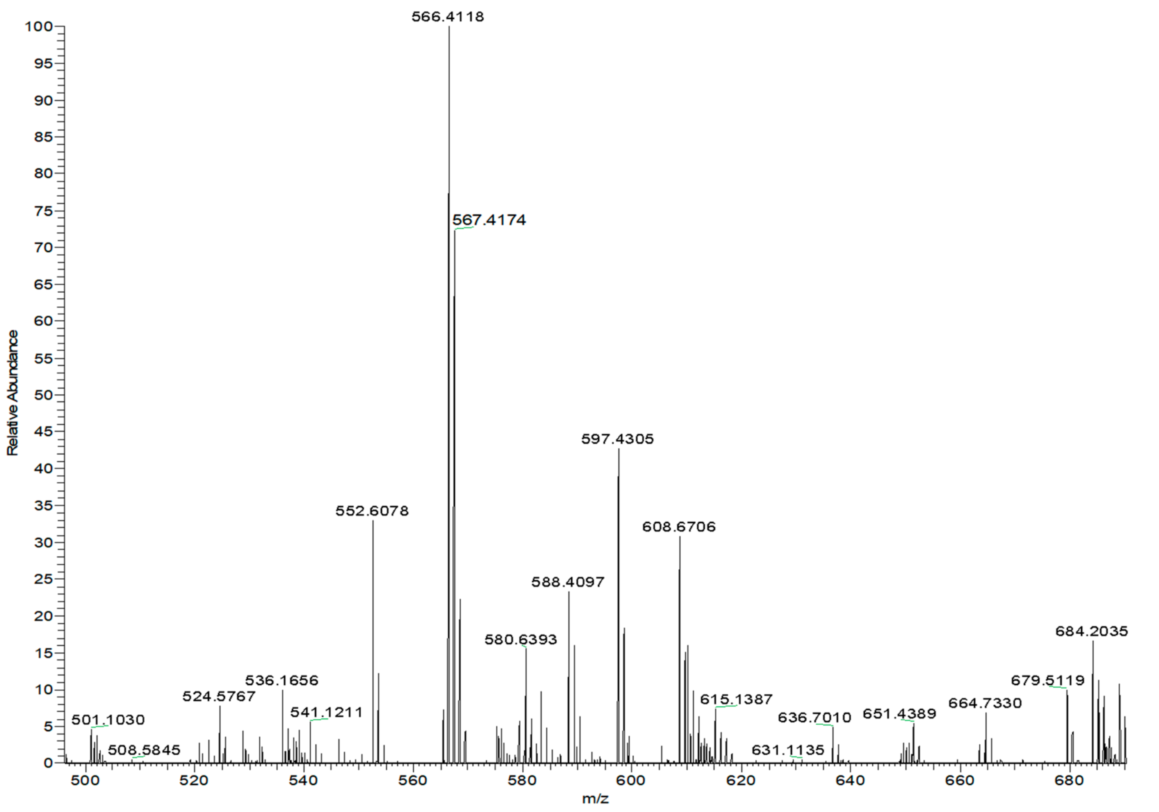

Figure S3. HRMS spectrum of the purified diatoxanthin (in CH_3OH).

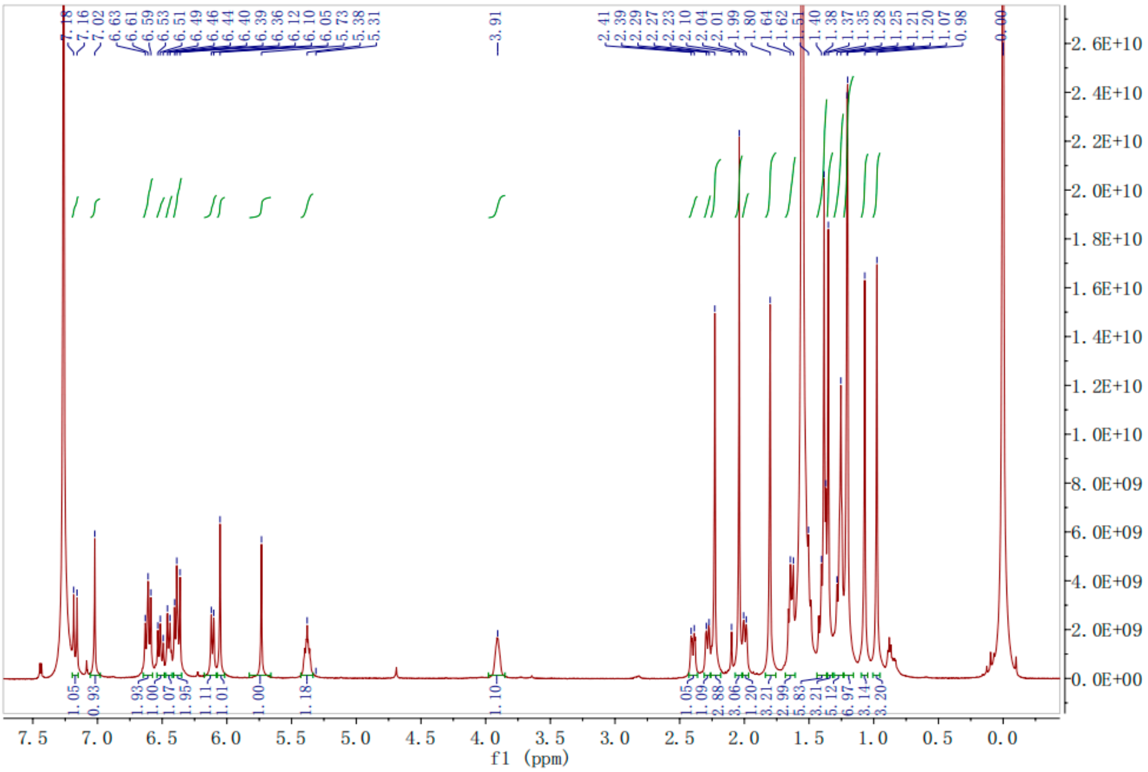

Figure S4. ^{1}H NMR spectrum (600 MHz) of the purified peridinin (in $CDCl_3$).

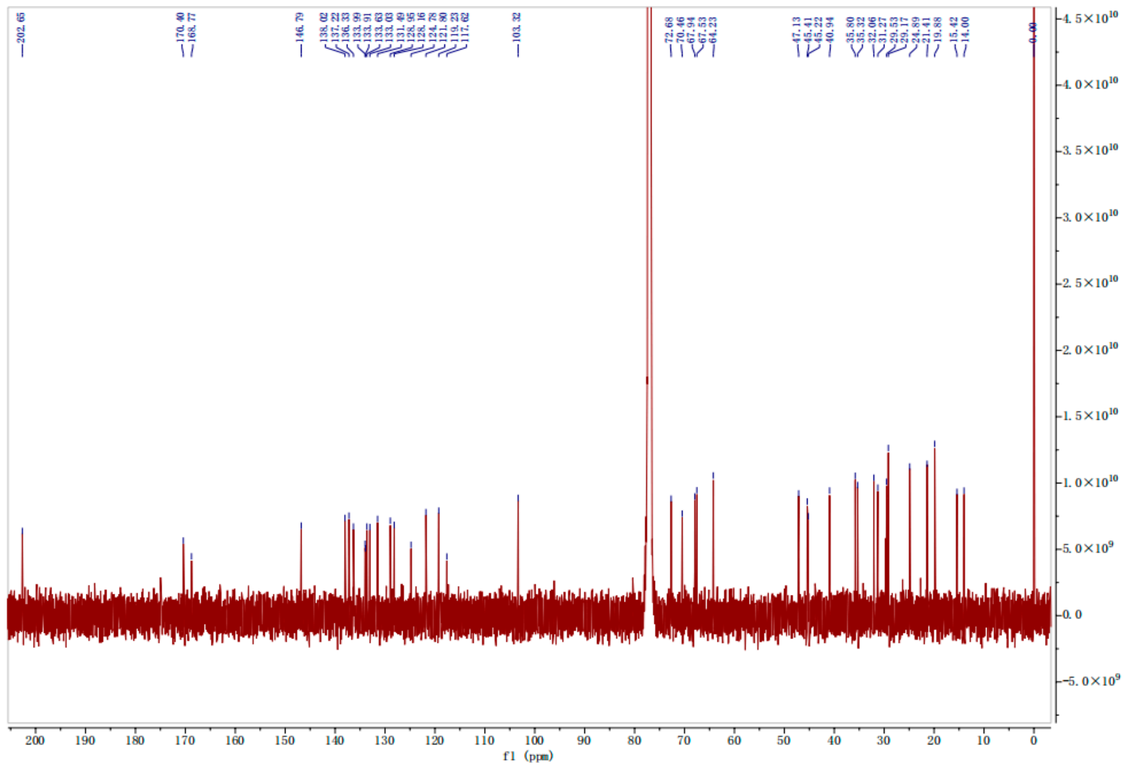

Figure S5. ^{13}C NMR spectrum (150 MHz) of the purified peridinin (in $CDCl_3$).

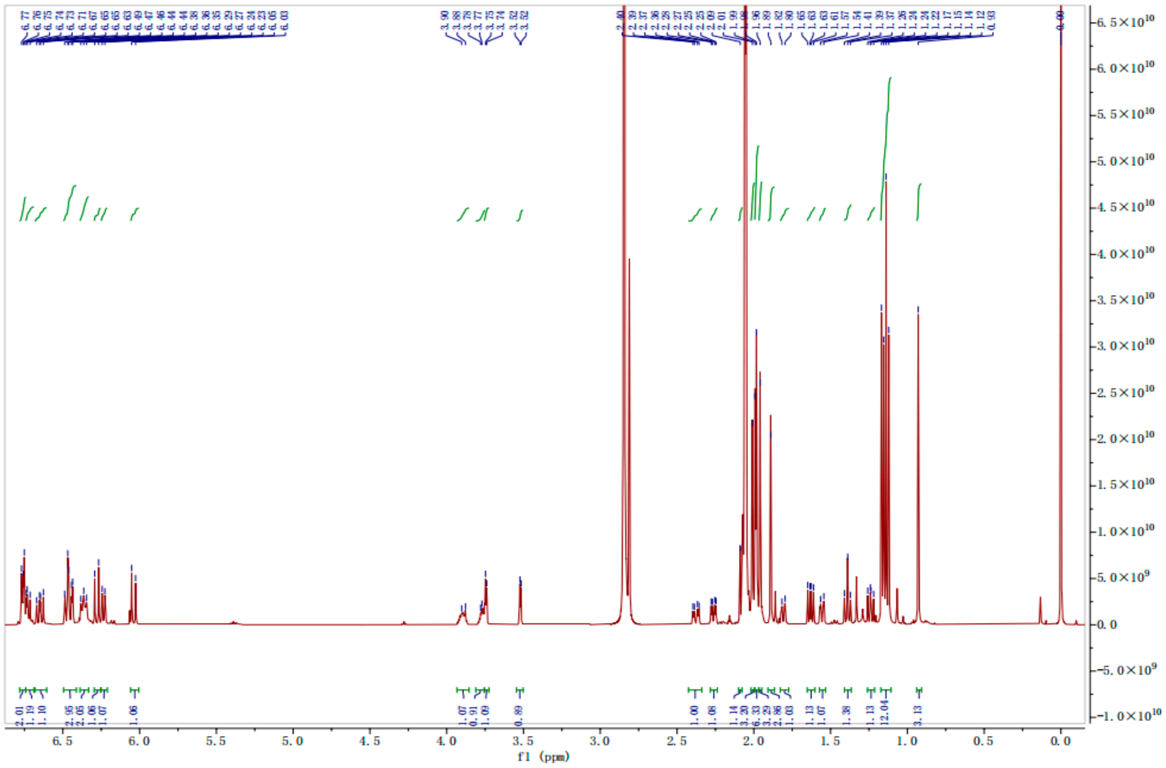

Figure S6. ^{1}H NMR spectrum (600 MHz) of the purified diadinoxanthin (in CD_3COCD_3).

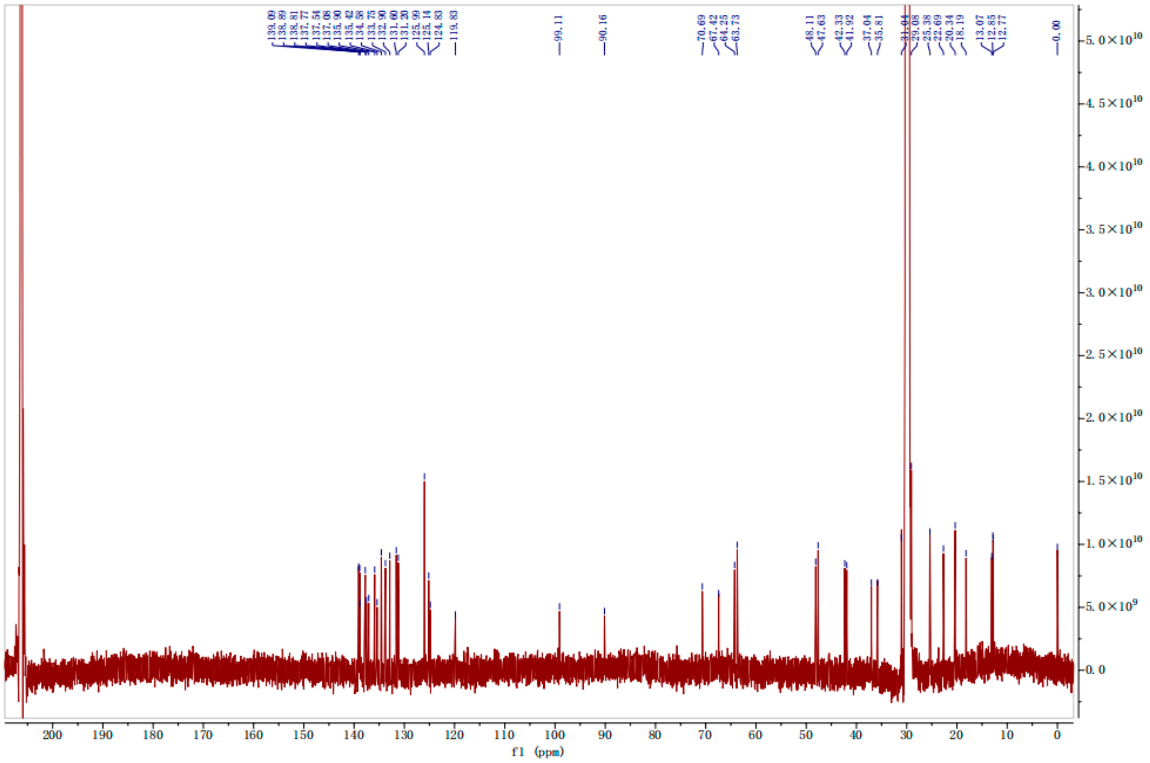

Figure S7. ^{13}C NMR spectrum (150 MHz) of the purified diadinoxanthin (in CD_3COCD_3).

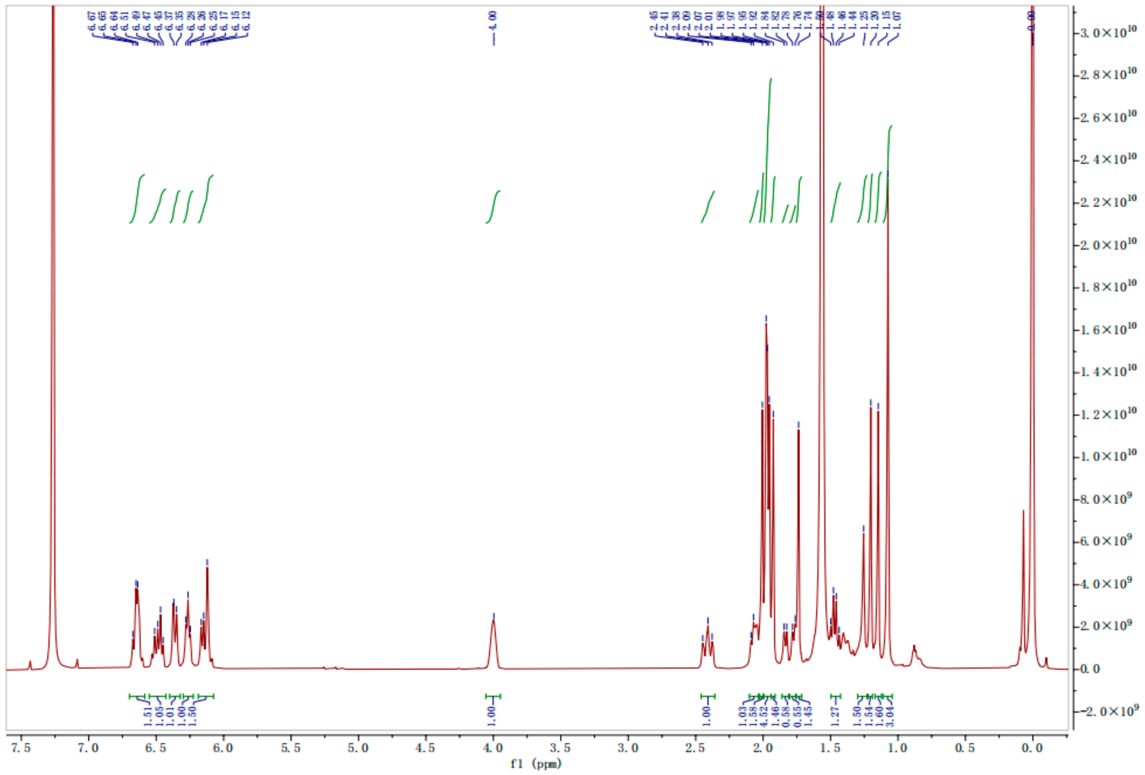

Figure S8. ^{1}H NMR spectrum (600 MHz) of the purified diatoxanthin (in $CDCl_3$).

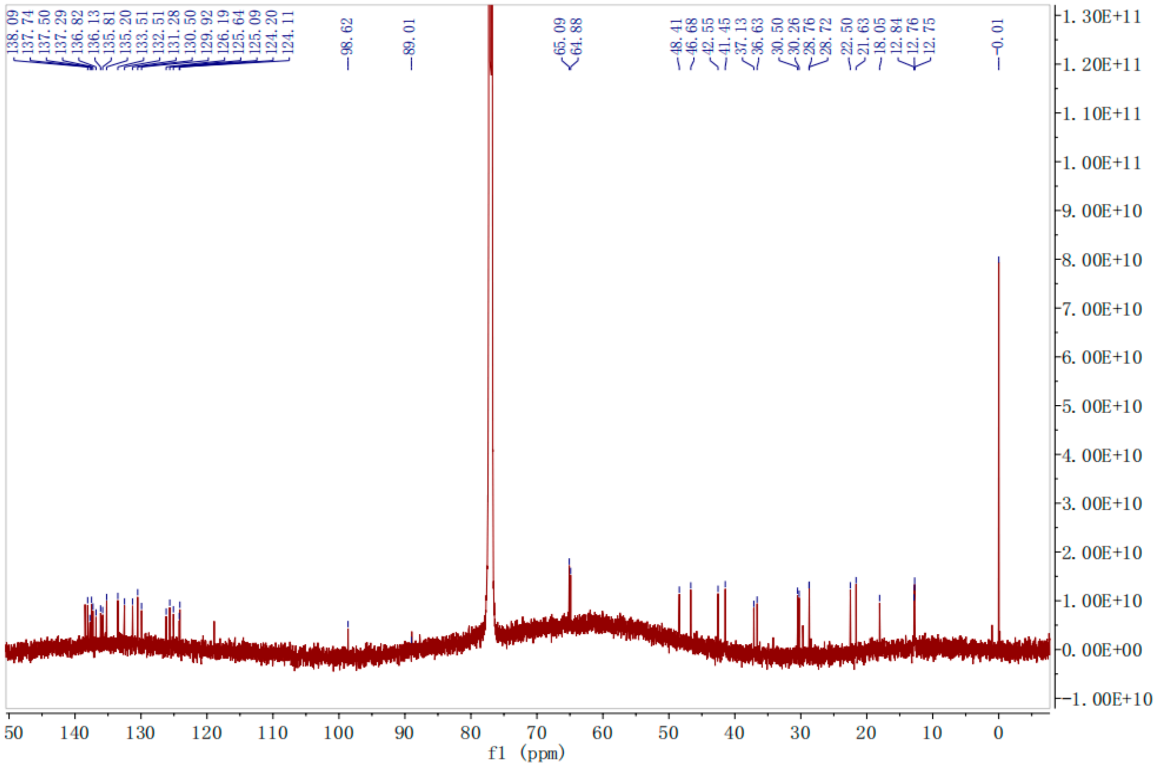

Figure S9. ^{13}C NMR spectrum (150 MHz) of the purified diatoxanthin (in $CDCl_3$).

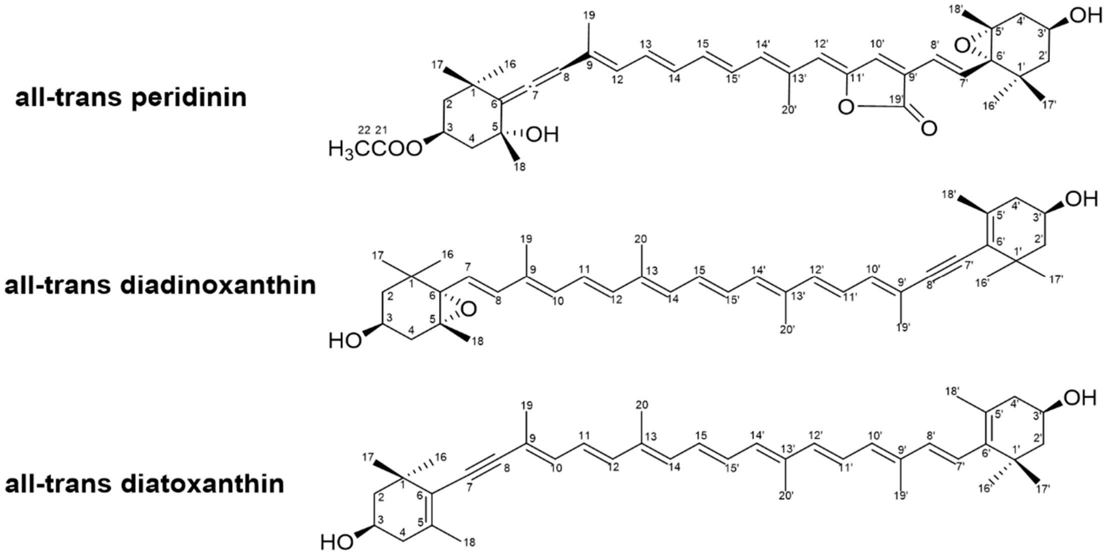

Figure S10. Structures of the purified all-*trans* peridinin, all-*trans* diadinoxanthin, and all-*trans* diatoxanthin isolated from *Amphidinium carterae.*

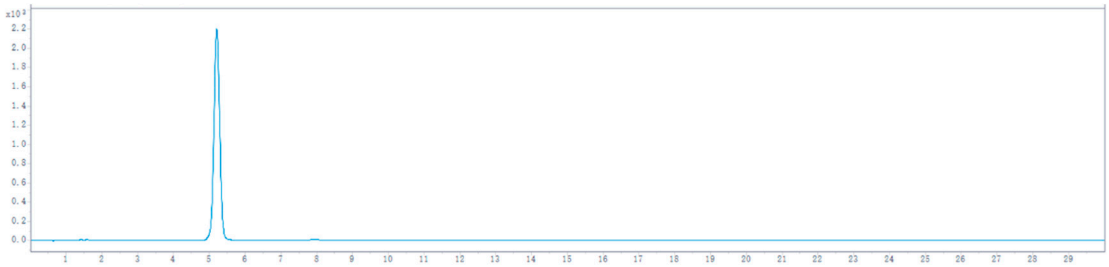

Figure S11. HPLC chromatogram (DAD, 450 nm) of the purified all-*trans* peridinin.

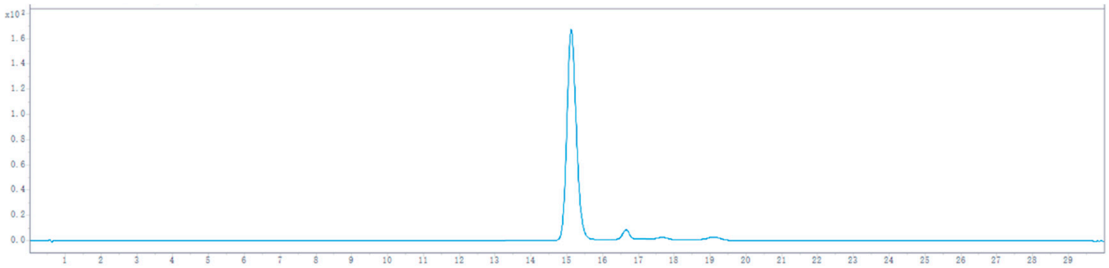

Figure S12. HPLC chromatogram (DAD, 450 nm) of the purified all-*trans* diadinoxanthin.

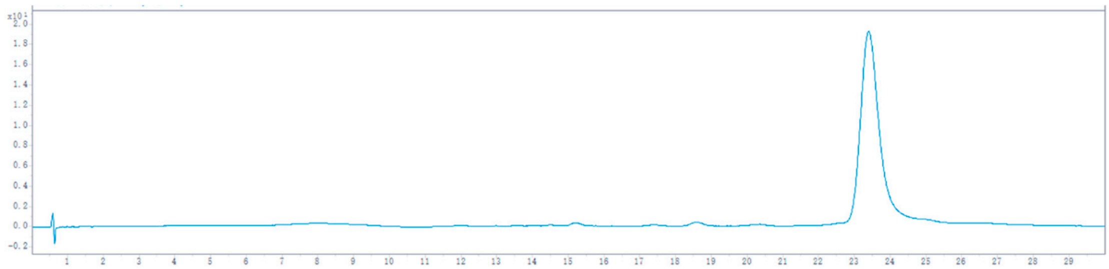

Figure S13. HPLC chromatogram (DAD, 450 nm) of the purified all-*trans* diatoxanthin.

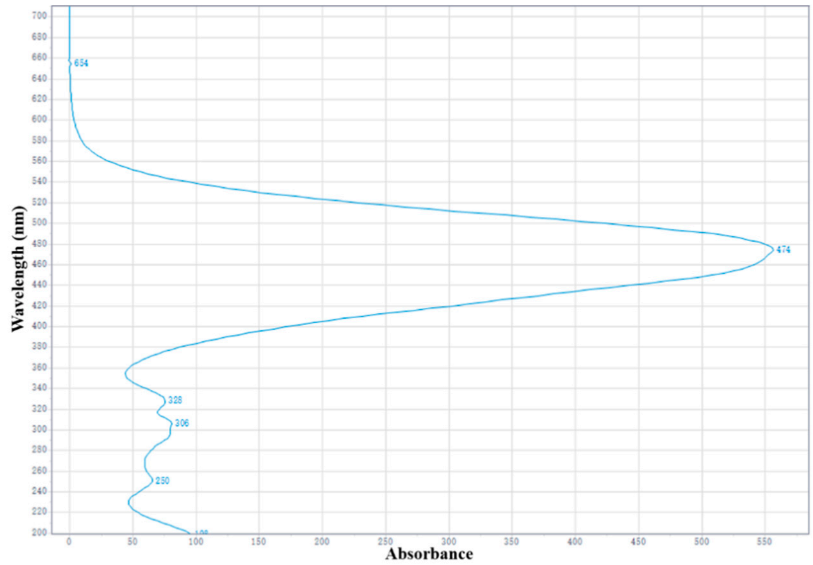

Figure S14. Absorption spectrum of the purified all-*trans* peridinin recorded during HPLC-DAD analysis.

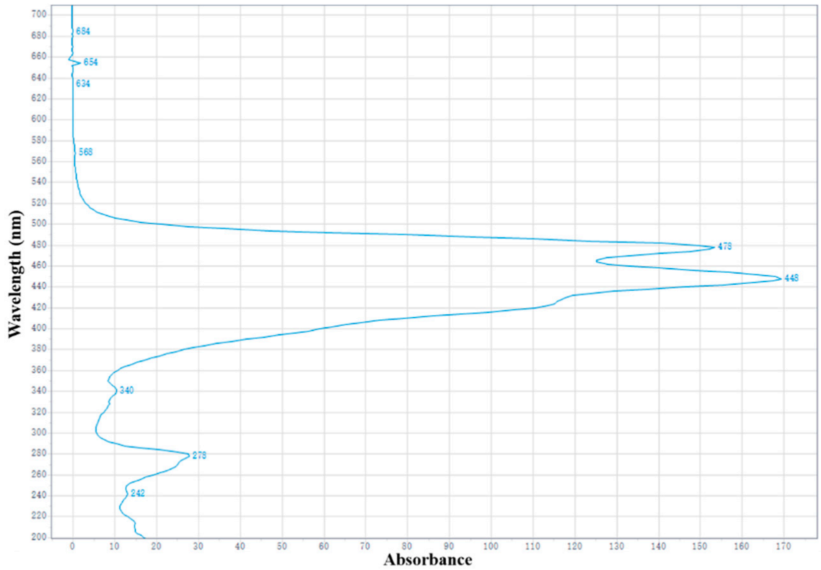

Figure S15. Absorption spectrum of the purified all-*trans* diadinoxanthin recorded during HPLC-DAD analysis.

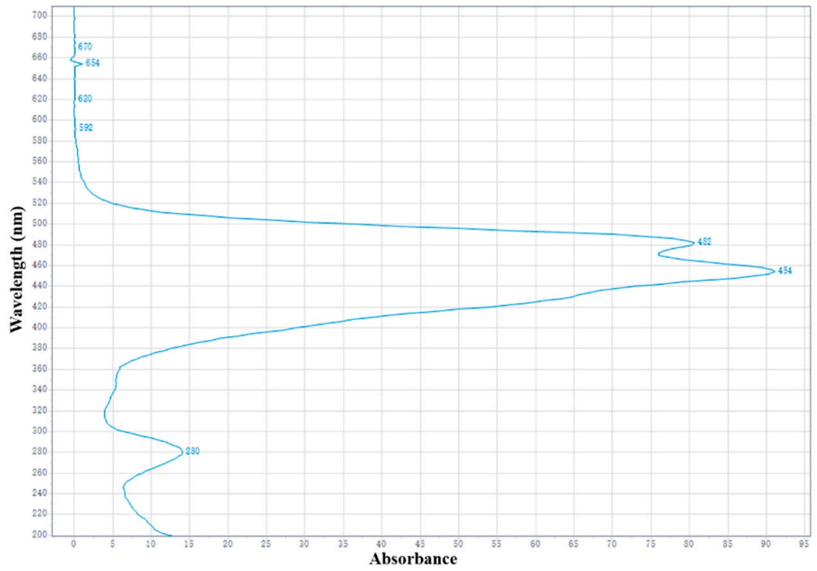

Figure S16. Absorption spectrum of the purified all-*trans* diatoxanthin recorded during HPLC-DAD analysis.

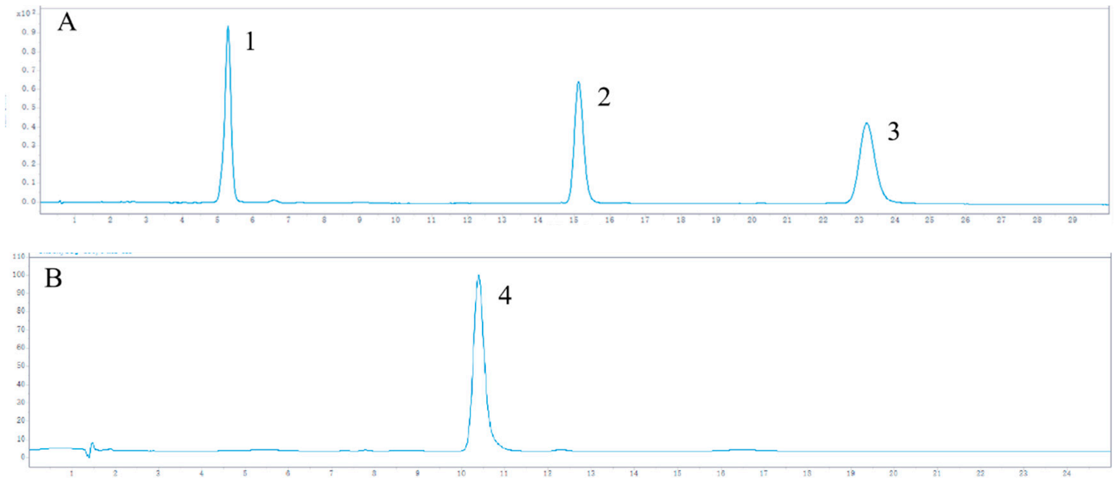

Figure S17. HPLC chromatograms of four pigments standards; (A) Individual peaks are 1. peridinin, 2. diadinoxanthin, 3. diatoxanthin; (B) Individual peak is 4. Chlorophyll a.

Table S1. ^{1}H NMR data for purified peridinin, diadinoxanthin, and diatoxanthin[a].

Position	Peridinin	Diadinoxanthin	Diatoxanthin
	^{1}H (multiplicity, J = Hz, 600 MHz)	^{1}H (multiplicity, J = Hz, 600 MHz)	^{1}H (multiplicity, J = Hz, 600 MHz)
1’			
2’	1.25 (1H, m)	1.81 (1H, J = 12.6 Hz, br d)	1.77 (1H, m)
	1.64 (1H, m)	1.39 (1H, J = 12.0, 12.0 Hz, dd)	1.48 (1H, m)
3’	3.91 (1H, m)	3.90 (1H, m)	4.00 (1H, m)
4’	1.64 (1H, m)	2.38 (1H, J = 17.4, 4.8 Hz, dd)	2.39 (1H, m)
	2.40 (1H, m)	2.03 (1H, m)	2.04 (1H, m)
5’			
6’			
7’	7.17 (1H, J = 15.6 Hz, d)		6.13 (1H, J = 15.8 Hz, d)
8’	6.37 (1H, J = 15.6 Hz, d)		6.10 (1H, J = 15.8 Hz, d)
9’			
10’	7.02 (1H, s)	6.48 (1H, J = 12.0 Hz, d)	6.16 (1H, J = 11.1 Hz, d)
11’		6.65 (1H, J = 15.0, 11.4 Hz, dd)	6.66 (1H, m)
12’	5.73 (1H, s)	6.45 (1H, m)	6.37 (1H, m)
13’			
14’	6.45 (1H, J = 11.5 Hz, d)	6.34 (1H, br d)	6.25 (1H, J = 10.4 Hz, d)
15’	6.61 (1H, m)	6.76 (1H, m)	6.63 (1H, m)
16’	0.98 (3H, s)	1.12 (3H, s)	1.07 (3H, s)
17’	1.21 (3H, s)	1.17 (3H, s)	1.07 (3H, s)
18’	1.20 (3H, s)	1.89 (3H, s)	1.74 (3H, s)
19’		2.01 (3H, s)	1.98 (3H, s)
20’	2.23 (3H, s)	1.99 (3H, s)	1.97 (3H, s)
1			
2	1.40 (1H, m)	1.57 (1H, J = 12.6 Hz, br d)	1.84 (1H, m)
	1.99 (1H, m)	1.23 (1H, m)	1.45 (1H, m)
3	5.38 (1H, m)	3.77 (1H, m)	4.00 (1H, m)
4	1.50 (1H, m)	1.63 (1H, J = 14.4, 8.7 Hz, dd)	2.43 (1H, m)
	2.28 (1H, m)	2.26 (1H, J = 14.4, 5.1 Hz, dd)	2.07 (1H, m)
5			
6			
7		6.04 (1H, J = 15.0 Hz, d)	
8	6.05 (1H, s)	6.28 (1H, J = 15.6 Hz, d)	
9			
10		6.24 (1H, J = 11.4 Hz, d)	6.46 (1H, J = 11.8 Hz, d)
11		6.71 (1H, m)	6.51 (1H, m)
12	6.11 (1H, J = 11.6 Hz, d)	6.45 (1H, m)	6.35 (1H, m)
13	6.60 (1H, m)		
14	6.38 (1H, m)	6.38 (1H, br d)	6.27 (1H, J = 10.7 Hz, d)
15	6.52 (1H, m)	6.76 (1H, m)	6.63 (1H, m)
16	1.38 (3H, s)	1.15 (3H, s)	1.15 (3H, s)
17	1.07 (3H, s)	0.93 (3H, s)	1.20 (3H, s)
18	1.35 (3H, s)	1.14 (3H, s)	1.92 (3H, s)
19	1.80 (3H, s)	1.98 (3H, s)	2.01 (3H, s)
20		1.96 (3H, s)	1.95 (3H, s)
21			
22	2.04 (3H, s)		

[a] Spectra were recorded at 600 MHz for ^{1}H NMR using TMS signals as the internal standard. The purified peridinin and diatoxanthin were dissolved in $CDCl_3$, respectively. The purified diadinoxanthin was dissolved in CD_3COCD_3.

Table S2. ^{13}C NMR data for purified peridinin, diadinoxanthin, and diatoxanthin[a].

Position	Peridinin	Diadinoxanthin	Diatoxanthin
	^{13}C (150 MHz)	^{13}C (150 MHz)	^{13}C (150 MHz)
1’	35.3	37.0	37.1
2’	47.1	47.6	48.4
3’	64.2	64.3	65.1
4’	40.9	42.3	42.5
5’	67.5	138.8	126.2
6’	70.5	124.8	137.7
7’	133.6	90.2	125.6
8’	121.8	99.1	138.5
9’	124.8	119.8	135.8
10’	136.3	135.9	131.3
11’	146.8	125.1	125.1
12’	119.2	138.9	137.5
13’	133.9	137.1	136.8
14’	138.0	133.8	132.5
15’	131.5	131.2	129.9
16’	24.9	29.1	28.7
17’	29.5	31.0	30.3
18’	19.9	22.7	21.6
19’	168.8	18.2	12.8
20’	15.4	12.8	12.8
1	35.8	35.8	36.6
2	45.4	48.1	46.7
3	67.9	63.7	64.9
4	45.2	41.9	41.5
5	72.7	67.4	137.3
6	117.6	70.7	124.2
7	202.7	126.0	89.0
8	103.3	137.8	98.6
9	134.0	135.4	118.9
10		132.9	135.2
11		126.0	124.1
12	128.2	139.1	138.1
13	129.0	137.5	136.1
14	133.0	134.6	133.5
15	137.2	131.6	130.5
16	29.2	25.4	28.8
17	32.1	31.0	30.5
18	31.3	20.3	22.5
19	14.0	13.1	18.1
20		12.9	12.8
21	170.4		
22	21.4		

[a]Spectra were recorded at 150 MHz for ^{13}C NMR using TMS signals as the internal standard. The purified peridinin and diatoxanthin were dissolved in $CDCl_3$, respectively. The purified diadinoxanthin was dissolved in CD_3COCD_3.
